# Mesolithic hearth-pits and formation processes: a geoarchaeological investigation of sediments from El Arenal de la Virgen site (SE Iberia)

**DOI:** 10.1007/s12520-023-01794-5

**Published:** 2023-06-22

**Authors:** Ana Polo-Díaz, Jose Ramón Rabuñal, Guillaume Guérin, Javier Fernández-López de Pablo

**Affiliations:** 1 Departamento de Geografía, Prehistoria y Arqueología, Universidad del País Vasco/Euskal Herriko Unibertsitatea UPV/EHU, Vitoria-Gasteiz, Spain; 2 I.U. de Investigación en Arqueología y Patrimonio Histórico, Universidad de Alicante, Alicante, Spain; 3 Department of Archaeology and Heritage Studies, Aarhus University, Moesgård Allé 20, 8270 Højbjerg, Denmark; 4 Univ Rennes, CNRS, Géosciences Rennes, UMR 6118, 35000 Rennes, France

**Keywords:** Mesolithic, Hearth-pits, Geoarchaeology, Formation process, Iberian Peninsula

## Abstract

Hearth-pits are some of the most common archaeological features documented in open-air Mesolithic sites, especially in coversand areas of NW Europe. However, very few geoarchaeological studies have addressed their formation, function and relationship with occupation surfaces. This work introduces new interdisciplinary investigations on the sediments of the Mesolithic open-air site of El Arenal de la Virgen (SE Iberia). A selection of five hearth-pits from two different occupation phases (Phase 1: 9.3–9.1 cal ka BP and Phase 2: 8.6–8.3 cal ka BP) has been analysed using stratigraphy, texture, soil chemistry, micromorphology, petrography and OSL and TL analyses. Combustion traits of the carbonate rock assemblages preserved in the sediments of the hearth-pits have also been investigated and compared to reference and experimental data from local geogenic materials. Our results allowed us to discuss the anthropogenic origin and taphonomy of the hearth-pits studied and approach their function. The structures from Phase 1 are interpreted as a possible oven and a dumping feature linked to single/occasional use events. In contrast, for hearth-pits from Phase 2, we propose they were related to combustion and dwelling areas subject to recurrent occupation episodes and disturbance. Finally, our sedimentary and soil data revealed existing favourable paleoenvironmental conditions during the Mesolithic occupation of the site characterized by increased moisture, temperature and vegetation cover, in contrast to the Pleistocene and Middle Holocene periods pre- and post-dating the human settlement. This work highlights the potential of integrating geoarchaeological and contextual evidence to clarify the factors involved in the formation of hearth-pits and infer intra-site occupation patterns.

**Supplementary information:**

The online version contains supplementary material available at 10.1007/s12520-023-01794-5.

## Introduction

Hearth-pits are a major source of archaeological evidence of open-air Mesolithic sites in European coversand regions. These shallow excavated features present a characteristic dark, organic-rich, sedimentary infill, and contain artefacts, charcoal and plant remains employed for the reconstruction of subsistence activities, paleoenvironments and chronological framing of Early Holocene hunter-gatherers (Crombé 2005; Fries et al. 2013; Gehlen et al. 2020).

Their function and possible activity area organisation have traditionally been inferred from the spatial distribution of associated archaeological features (Peeters and Niekus 2017) and ethnographic analogy (Mithen and Wicks 2018). Nevertheless, no detailed and comprehensive study of hearth-pits has hitherto been conducted, despite calls to clarify their origin (Crombé et al. 2015). Regarding this latter issue, a controversy has recently emerged. Some studies have linked the origin of hearth-pits to the use of fire in human activities, such as hazelnut roasting or tar preparation (Huisman et al. 2019, 2020). Others, on the other hand, have attributed their formation to collapsed bioturbation features affected by wildfires (Crombé and Langohr 2020a, b). The discussion has been focused on the examination of the sediment and soil study of some of these features, raising the need to further explore the potential of high-resolution analyses (Huisman and Tebbens 2021). Yet it is also necessary to consider sedimentary data in its wider archaeological context in order to test Early Holocene hearth-pit formation hypotheses.

The Iberian Peninsula presents the potential to address this question, expanding the regional focus of the geoarchaeological investigation.

An increasing number of Mesolithic combustion features has been recorded over the past two decades in this area — exemplified by Cabezo de la Cruz (Rodanés and Picazo 2013), Font del Ros (Martinez-Moreno and Mora 2011), Parque Darwin (Escobar 2010), Benàmer (Torregrosa Giménez et al. 2011) and Casa Corona (Fernández-López de Pablo et al. 2013). However, the recognition of fire traces has not been accompanied by specific contextual geoarchaeological studies, and the conclusions about the presumed function of these features have largely relied on archaeobotanical data (Badal 2013; Roda-Gilabert et al. 2013; Berihuete Azorín, et al. 2017).

New excavations at the open-air Mesolithic campsite of El Arenal de la Virgen, in the Upper Vinalopó Valley (SE Iberian Peninsula), have provided the opportunity to address this issue, and to contribute to current discussions on hearth-pit formation.

The present paper shares the results of the study of these structures documented at the site. The main goals of our research are summarized as follows:

1. To establish a specific methodological framework based on the integration of multiproxy parameters at various scales in order to investigate Mesolithic dwellings with complex formation dynamics located on coversand deposits.
2. To analyse the mineral and organic composition of the hearth-pit infills to characterise the sediments, the combustion traits and the degree of thermal impact.
3. To clarify the role of anthropogenic and natural dynamics on the accumulation and alteration of hearth-pit deposits and to discuss structure formation within its archaeological context, so as to reconstruct the human occupation of the site.

## El Arenal de la Virgen site

**Fig. 1:**
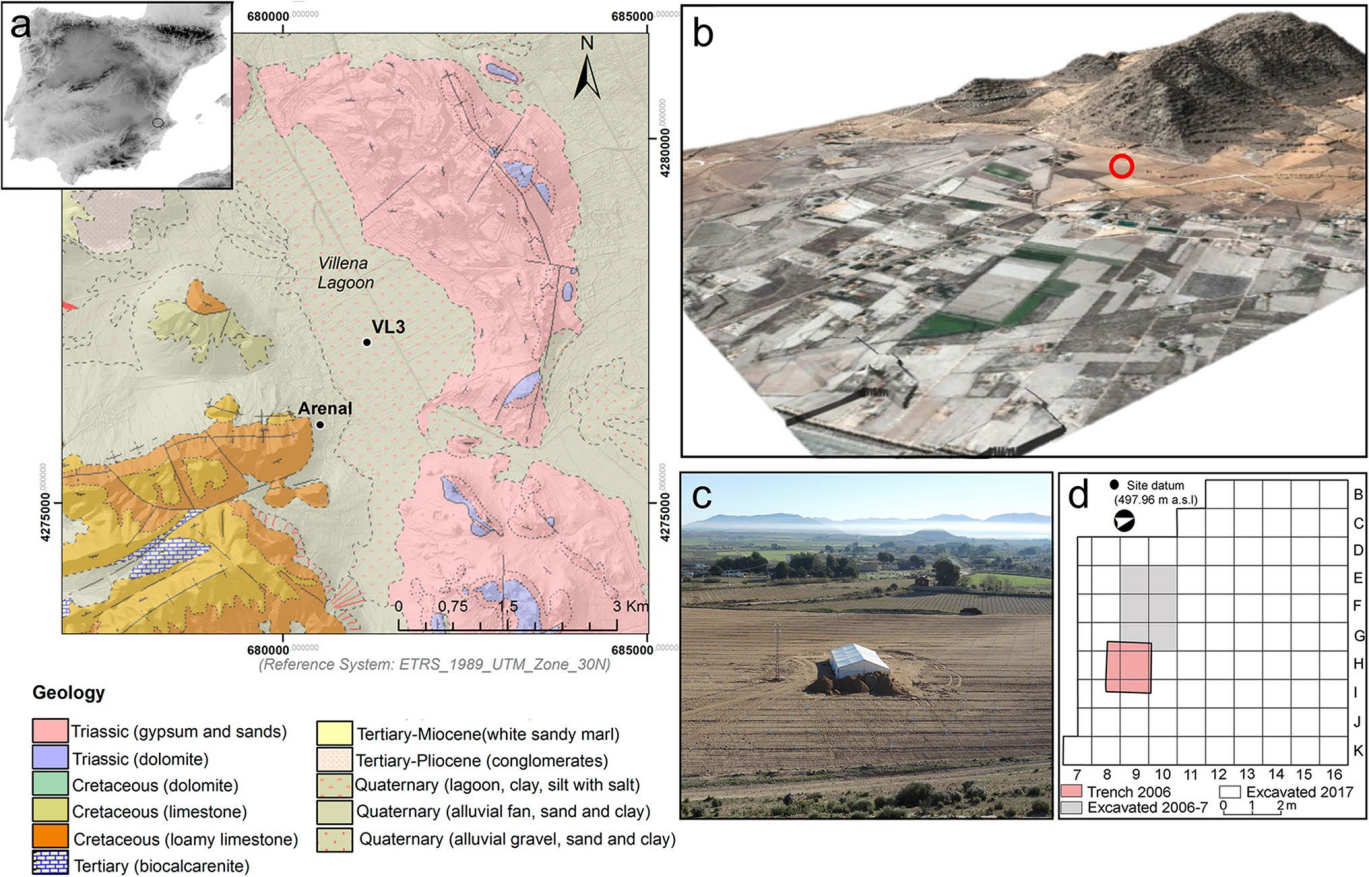
El Arenal site and its geological and geomorphological setting. **a** Geological map of the site indicating its location at the foot of Sierra del Castellar and next to the Villena paleolake. **b** Location of the site at the foot of Sierra del Castellar. View from the north. **c** Excavation area from the south. **d** Excavation grid showing the extension of the site work overtime

El Arenal de la Virgen (hereafter, El Arenal) is an open-air site located on coversand deposits in the Upper Vinalopó Basin, at 503 m.a.s.l. on the SW shore of the Villena Lagoon (38° 36′ 55.055″ lat, 0° 55′ 36.31′). This lagoon is a paleolake that was drained at the beginning of the nineteenth century, but was active however, during the Late Pleistocene (Jones et al. 2018). The geology of the area is dominated by Cretacic limestones, Triassic gypsum clays and Tertiary conglomerates and calcarenites. Quaternary glacis formations and alluvial fans accumulate at the margins of the paleolake, together with significant wind-blown deposits (IGME MAGNA50_845 sheet) (Fig. 1a). The latter form aeolian sheets that constitute the substrate of the Mesolithic archaeological deposits. The site is currently under a Mediterranean climate with Continental traits, resulting from its location midway between the Mediterranean Rim and the Iberian Central Plateau. Maximum precipitations take place in spring and summer with a mean annual precipitation of 359 mm, and the vegetation cover is dominated by the *Quercus* species.

Local soils are characterised by sandy substrates in which calcitic and gypsic formation processes linked to arid/semiarid conditions are dominant (Torre and Alías Pérez 1996).

**Fig. 2:**
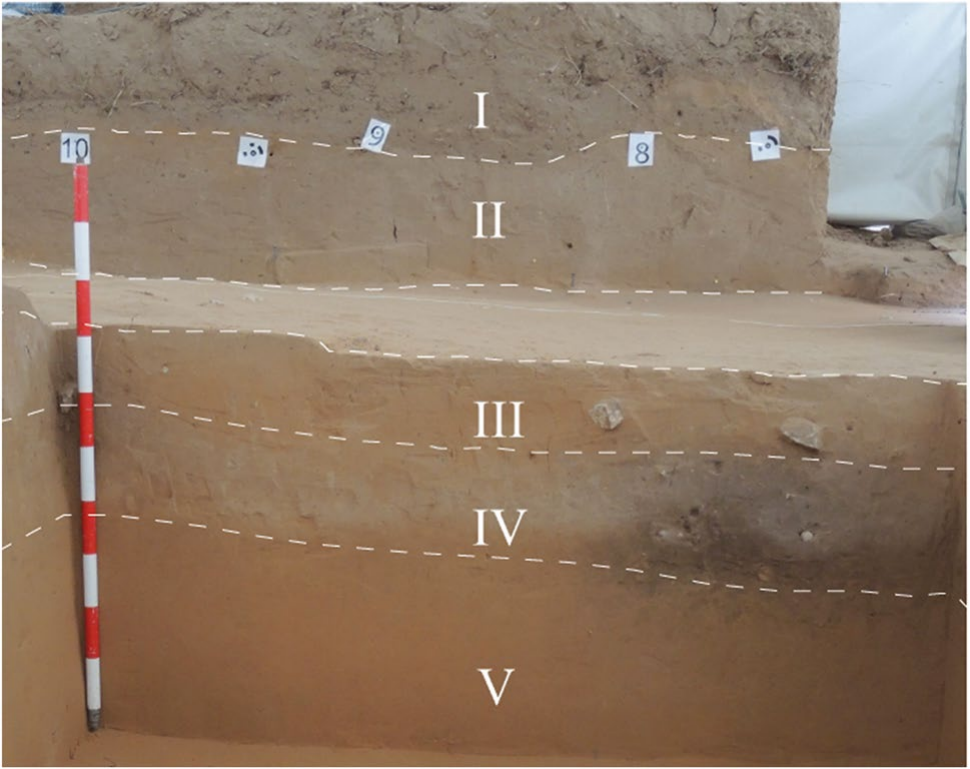
El Arenal. E profile. Detail of the stratigraphic sequence showing Units V, IV and III in the area where the test trench was excavated in 2006. Units II and I were uncovered during the works conducted in 2017

Archaeological excavations at El Arenal site started in 2006 as part of a test trenching programme that uncovered Early Holocene archaeological deposits (Fernández-López de Pablo et al. 2008). These works were further expanded in 2007, ultimately covering a total excavation area of 6 m^2^. A succession of five main sedimentary units was documented at the site (Fig. 2). Details and correlations of the full stratigraphy of the site are presented elsewhere (Fernández-López de Pablo et al. 2023; Fernández-López de Pablo et al. 2011a). Unit IV, radiocarbon dated to 7750 ± 40 BP (8575–8475 cal BP), revealed a hearth-pit that was spatially associated with lithic as well as land snail assemblages and thermo-altered limestone clasts (Fernández-López de Pablo et al. 2011a, b). Fieldwork resumed in 2017. At this time, the original excavation area notably increased — reaching 84 m^2^ — (Fig. 1d, c) together with the archaeological evidence, including the number of hearth-pits (SU structures) which were recorded exclusively in Unit IV.

**Fig. 3:**
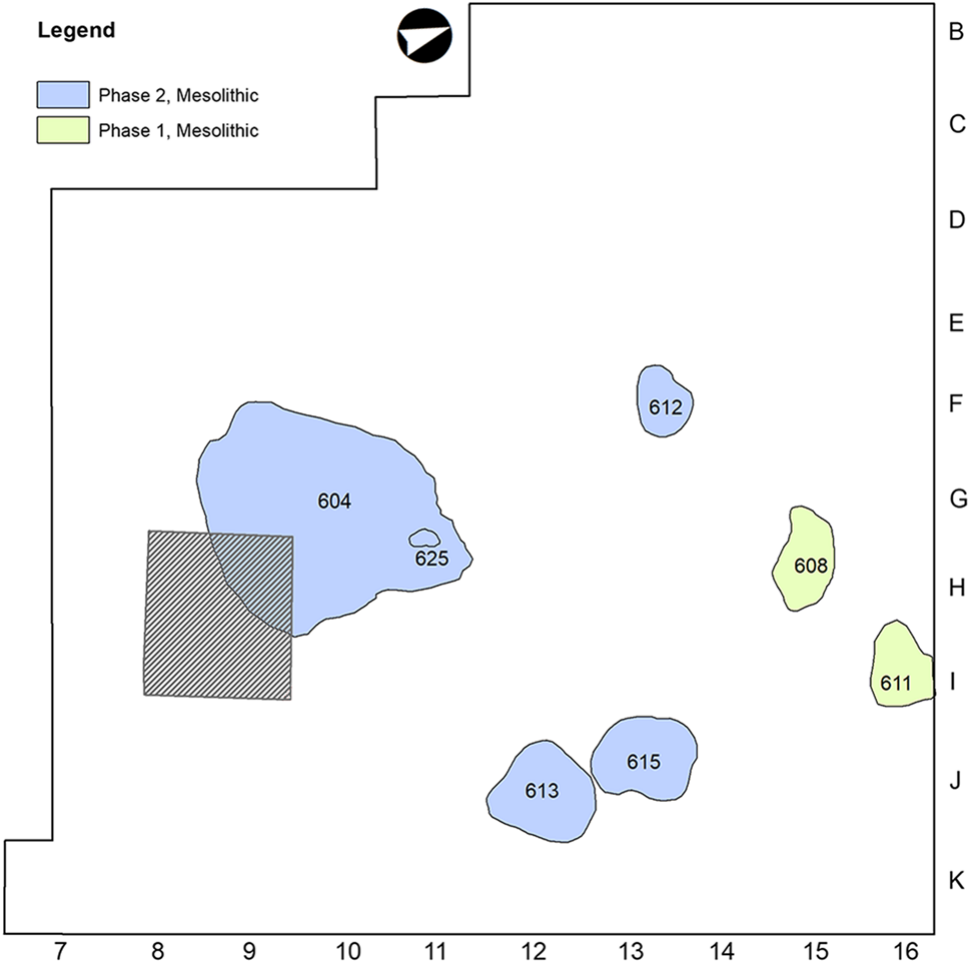
Plan of El Arenal site showing the spatial distribution and chronological phasing of the hearth-pits investigated in this study. The shaded square represents the test trench excavated in 2006

The results of the 2017 field session confirmed the Early Mesolithic attribution of the lithic assemblages of Unit IV, providing a more refined spatial and chronological picture of the Mesolithic occupation of the site (Fernández-López de Pablo et al. 2023). As discussed later (see the “Sediments and formation process of Unit IV” section), microstratigraphic evidence indicated a sedimentary hiatus within Unit IV, consistent with the new radiocarbon dates yielded by charcoal fragments and pinecone scales recovered from the recently excavated hearth-pits at the site. Bayesian modelling of these stratigraphically constrained radiocarbon dates generated two occupation phases associated to Unit IV: Phase 1 spans 9.3–9.1 cal ka BP, linked to structures SU 608 and SU 611 at the north excavation area; Phase 2, for its part, spans 8.6–8.3 cal ka BP and concentrates most of the lithic assemblages and occupation features in structures SU 604, SU 625, SU 613, SU 615, SU 627 and SU 612 (Fig. 3). Such structures closely resemble hearth-pits and were documented along with other latent combustion loci identified via the spatial analysis of thermo-altered chipped-stone assemblages (Rabuñal 2021).

## Materials and methods

To meet our research objectives, sediment infills and rock assemblages from a selection of presumed hearth-pits documented at El Arenal were analysed together with geological samples from the Sierra del Castellar outcrop (Fig. 1b). For the purpose of this work, we also studied the sediments of Unit IV, encasing the hearth-pits, in addition to the contacts between the overlying Unit III and the underlying Pleistocene sands of Unit V. Radiocarbon dating results of charcoal recovered from the hearth-pit sediment infills are provided in Table 1.

**Table 1:** Radiocarbon dataset and calibrated ^14^C radiocarbon dates (unmodelled posterior distributions) of El Arenal hearth-pits investigated in this study (modified after Fernández-López de Pablo et al. 2023). Radiocarbon dates have been calibrated using the Oxcal 4.4 (Bronk Ramsey 1994) and the Intcal 20 radiocarbon calibration curve (Reimer et al. 2020)

Unit	Phase	S.U./hearth-pit	Z	*Sample*	Lab Ref	BP	S	C13	cal a BP
IV	1	608/3	180–172	*Quercus sp*	Beta-493220	8220	30	− 27.9	9394–9027
IV	2	608/1	166–161	*Q. evergreen*	Beta-493219	7700	30	− 23.7	8547–8411
IV	1	611	170	*Q. sp evergreen*	Beta-473942	8200	30	− 26.4	9275–9026
IV	2	625	217	*Q. sp evergreen*	Beta-473944	7850	30	− 25.5	8765–8546
IV	2	613/1	184–187	*Q. evergreen*	Beta-493221	7820	30	− 25.4	8698–8520
IV	2	613/1	194–190	*Quercus sp*	Beta-493225	7770	30	− 25.8	8600–8449
IV	2	613/2	193–187	*Pinus cone scale*	Beta-493222	7660	30	− 28.1	8536–8389
IV	2	604	217	*Q. evergreen*	Beta-243772	7750	40	− 25.8	8595–8426
IV	2	604/4	220–216	*Pinus cone scale*	Beta-493226	7550	40	-	8420–8210
IV	2	612	187–194	*Quercus sp*	Beta-502077	7680	30	− 26.6	8541–8407
IV	2	615	191	*conifer*	Beta-493224	7480	30	-	8371–8195

The investigation presented here is based on a geoarchaeological multiproxy approach encompassing stratigraphy, micromorphology and petrography, textural analyses, soil chemistry and luminescence (both optically stimulated luminescence, or OSL, and thermoluminescence, or TL). Data analysed comprise results obtained from hearth-pit sediments, reference material produced, experimental burning of carbonate rocks and field observations.

Relevant available data on the spatial analysis of burnt lithic assemblages and archaeobotanical remains associated with the hearth-pits were also integrated into the discussion together with chronological and paleoenvironmental frameworks of the site (Fernández-López de Pablo et al. 2023 and Rabuñal 2021).

### Micromorphology and petrography of archaeological and reference samples

To perform the micromorphological analysis, we studied 30 large (13 × 5.5 cm) thin sections from intact sediment block samples documenting the stratigraphy and the sedimentary context of the selected hearth-pits corresponding to Mesolithic Phase 1 (SU 608 and SU 611) and Mesolithic Phase 2 (SU 604, SU 613, SU 615 and SU 625) (Fig. 2).

Plaster bandages and flexible aluminium moulds were used in the field to ensure that the blocks remained intact upon removal. In the laboratory, the samples were dried in an oven for several weeks at 25–30 °C and subsequently hardened using a mixture of resin, solvent and catalyst for 3–4 weeks.

Slides were scanned at high-resolution to allow mesoscopic descriptions of the microstructure and sediment feature distribution.

Carbonate rock assemblages are one of the most relevant components of hearth-pits at El Arenal, and their study can potentially help to clarify the origin and function of the structures they are associated with. Accordingly, intra and off-site samples were collected for macroscopic characterisation and petrographic analysis. First, a set of 25 carbonate rocks from the Upper Cretaceous outcrop of Sierra del Castellar, located next to the site (Fig. 1b), was collected and documented in detail. Second, 11 representative specimens were subsampled and processed into small thin sections. (4 × 3 cm) to provide local geogenic reference material. Three samples were selected for experimental burning to assess thermal impact on the carbonate material. Third, rock specimens from hearth-pits SU 604, SU 608, SU 613 and SU 611 were sampled, documented and processed into 4 small thin sections in order to characterise and compare them with the reference material and experimentally burned samples.

### Burning of carbonate rocks and petrography of experimental samples

The three experimental burning samples (Ref 4, Ref 8 and Ref 14) were selected based on representative features (i.e., morphology, size and alteration patterns) observed both in the archaeological and non-archaeological local limestones. The samples consisted of biosparitic limestone (Ref 4, Ref 8) and marl (Ref 14), whose macroscopic features were recorded before burning. Specimens Ref 8 and Ref 14 were subsequently cut into three sets to be burned separately for three hours (Soler 2003) in oxidising conditions in a muffle furnace at 300–350 °C, 500–550 °C and 700–750 °C. The aim was to document changes in geogenic carbonates with increasing temperature (Canti 2017). After completing the experiment, a total of 8 slides (4 × 3 cm) were produced for petrographic analysis. All samples were allowed to cool down gradually inside the furnace overnight after combustion, with the exception of 1 specimen burned at 500–550 °C (Ref. 8) which was removed immediately and left at room conditions (20–23 °C–70% RH approx.) to test the impact of thermal shock on the sample. Sample Ref. 4 was not sectioned prior to combustion in order to assess the thermal impact on the cortex and the total weight mass loss. This sample was also burned successively at 300–350 °C, 500–550 °C and 700–750 °C for 3 h allowing time to cool down between each of the burning episodes so as to document the accumulative effects of combustion.

Three criteria observed at macroscopic and microscopic levels were used to determine the thermal impact on the specimens: (1) the physico-mechanical changes (colour, fissure/fracture patterns, friability); (2) the degree of preservation of the crystalline structure; (3) the development of oxides.

A petrographic microscope equipped with a digital camera was used for the micromorphological and petrographic thin section analysis, applying a range of magnifications (× 20– × 400) and lights (plain polarized light: PPL) (crossed polarized light: XPL). Micromorphological descriptions followed international standards established in specialist literature (Stoops 2003, 2021).

### Texture and soil chemistry

A total of 30 bulk sediment samples were collected every 5 cm from the profiles of the hearth-pits SU 604; SU 613 and SU 625 and then processed for textural analysis and soil chemistry. We avoided removing carbonates from the samples processed for granulometry to prevent the distortion of grain size distributions. Sediments were separated into 9 textural classes by gravimetry to obtain a high-resolution textural pattern. We measured pH in 1:2.5 water solution (Dewis and Freitas 1970), and the organic matter (OM) was calculated using potassium dichromate as an oxidising agent for colorimetry (Walkley and Black 1934). Finally, the calcium carbonate equivalent was determined using a volumetric calcimeter.

### OSL and TL

The luminescence emitted by quartz and/or feldspar grains is the result of a radiative recombination of trapped electrons and holes in a crystalline mineral under stimulation. Thermoluminescence (TL) allows dating the last heating of a mineral (typically flint or quartz), while optically stimulated luminescence (OSL) targets the last exposure to light or heat in the past (Aitken 1985; Huntley et al. 1985; Murray et al. 2021).

In this study, however, we did not use luminescence to provide a site chronology. We sought to exploit changes in luminescence characteristics induced by heating. The most obvious example is the increase in sensitivity of quartz OSL resulting from heating to several hundred degrees Celsius (typically 700 °C); see Bøtter-Jensen (2000) especially Fig. 4.9. This characteristic is used to produce reference material for the calibration of laboratory radioactive sources used in luminescence dating (Hansen et al. 2015). Specifically, approximately 95% of quartz grains of this calibration quartz produce a detectable luminescence signal in response to a given dose of 4 Gy. Other studies have shown that repeated cycles of heating, irradiations and optical stimulations increase the quartz OSL sensitivity (e.g., Moska et al. 2010).

Another example of sensitised quartz comes from heating structures in the archaeological record, and more precisely from the Middle Palaeolithic site of Roc de Marsal. Many combustion structures, identified by the amount of stratified ash and reddened sediment, have been unearthed during recent excavations; the quartz retrieved from these structures, as well as from unheated sediment samples at the site, was dated with OSL. Having conducted single-grain OSL experiments, Guérin et al. (2017) observed that the fraction of quartz grains that emitted detectable OSL signals from heated samples was significantly larger (27–42%) than the same fraction from unheated samples (8–18%), despite some variability in both sample sets.

At El Arenal, 3 samples from hearth-pits SU 604, SU 608 and SU 611 were processed for single grain OSL and TL analyses. An additional set of 3 samples taken from different locations of the archaeological area outside the hearth-pit structures (base of Unit V, Unit III and Unit IV) was processed for reference. The objective was to detect the possible heating of the sediment. Contrary to sediment sampling for dating — during which exposure to light must be avoided — we simply carried a few grams of sediment away in plastic bags. It should be noted here that our aim in the present study is to test the potential of OSL and TL sensitivities to be used as proxies, among others, for past heating. As a side note, according to Zimmerman (1971), the sensitivity of quartz grains may be reduced by exposure to UV light. As a result, our hope was that this sensitivity change induced by light exposure would not be of sufficient magnitude to erase heat-induced differences between samples.

In the laboratory (IRAMAT, Bordeaux), the samples were first wet-sieved in order to isolate the 180–250 μm fraction. This fraction was subsequently treated with 10% HCl to remove carbonates and then, after rinsing, with concentrated H_2_O_2_ to remove organic material. The samples were later treated with 40% HF for 40 min to dissolve feldspar. After rinsing with HCl to eliminate fluorides, all fractions were sieved again at 180 μm. As a result, we obtained a quartz rich fraction.

A total of 300 grains obtained from each sample were then mounted on single-grain discs for individual stimulation with a green laser beam inside a Risø TL/OSL DA-20 reader (Bøtter-Jensen et al. 1999, 2010). Every disc was checked under a microscope after loading the single grains, to avoid — as much as possible — empty holes and/or pairs of grains in single holes. Even if such screening under a microscope does not completely exclude the presence of occasional empty holes and/or pairs of a grains in single holes, we estimate that between 95 and 100 holes were filled with at least one grain. Of these, we estimate that 1–5 holes might contain two grains. Given the proportion of grains emitting light (24% at most — see below in the results section), the probability of having two luminescent grains in one hole is small (less than 6%). Luminescence was detected using a photo-multiplier tube after passing through a 7.5-mm-thick Hoya U340 filter. After resetting any natural signal with OSL stimulation, all grains were delivered a ~ 10 Gy beta dose and measured after having been preheated at 220 °C.

To investigate the past heating of the sediment, we examined the fraction of grains that emitted detectable OSL (we only selected grains for which the uncertainty of the OSL signal, summed over the initial 0.05 s of stimulation, was less than 15%), and three other potential proxies: the intensity of the 110 °C TL peak during the preheating (i.e., prior to the OSL measurements), the total OSL signal intensity, summed over the 300 measured grains, and the median OSL signal among the grains that produced a detectable signal.

## Results

### The stratigraphy and sedimentary context of El Arenal hearth-pits

Unit IV has been documented across most of the excavation area except for the western section, where the impact of erosion (i.e., deflation and slope dynamics) involved in the accumulation of the overlying Units III and II prevented its preservation.

**Fig. 4:**
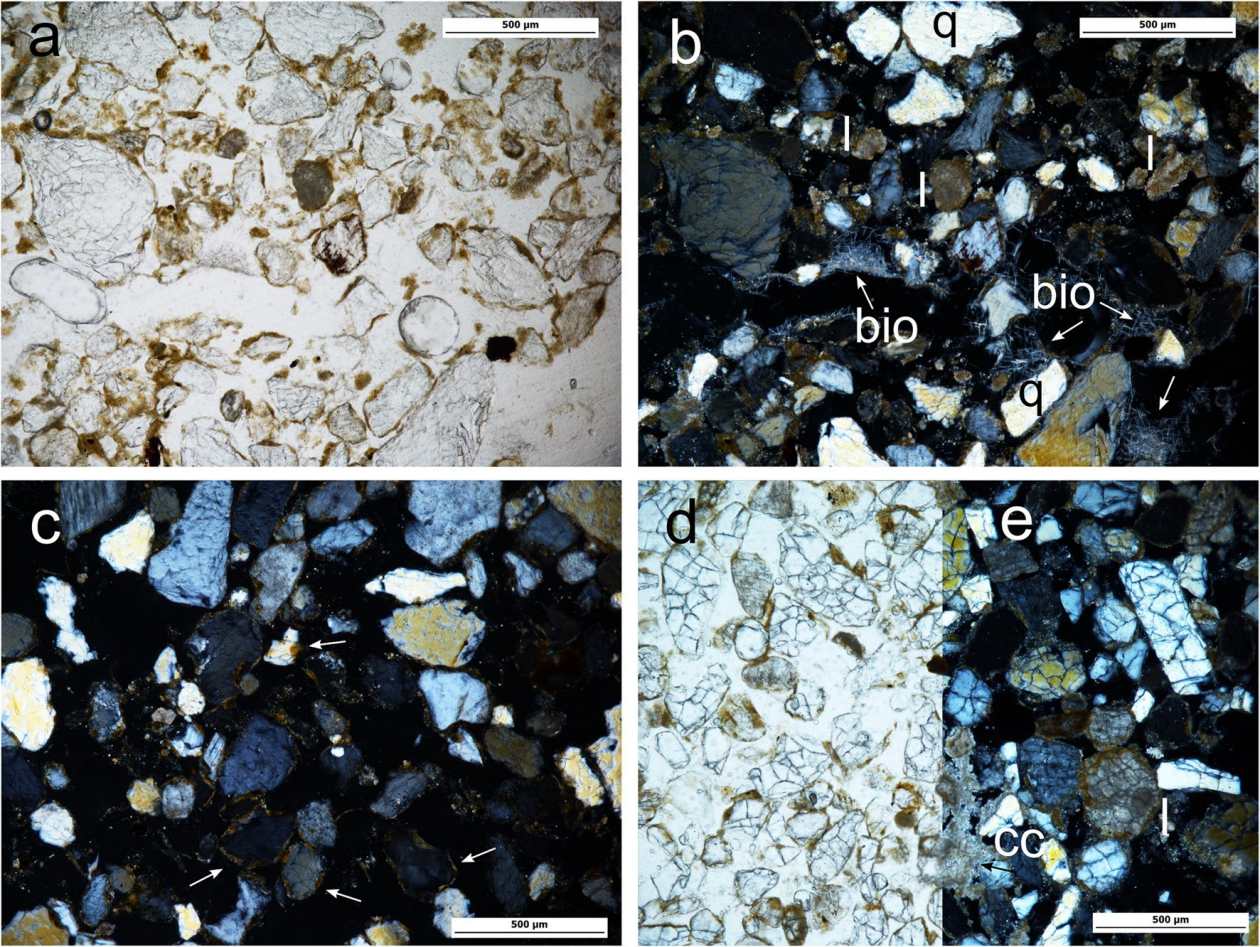
El Arenal. Sedimentary context of the hearth-pits studied (see Fig. 8 for location of samples illustrating this figure). Microscopic images from micromorphological thin sections. **a**, **b** Unit IV. PPL and XPL (AR-604–2). Sediment mainly consisting of quartz (q) and carbonate/limestone (l) sands. Note concentration of needle-fiber calcite (bio) related to fungi (Callot et al. 1985; Verrecchia and Verrecchia 1994) and channel from soil fauna activity. **c** Unit III (AR-604–1). XPL. Sediment matrix showing partial clay coatings preserved in the hollows of the quartz grain surface (arrows) and general lack of calcitic features. **d**, **e** Unit V. PPL and XPL (AR-604-12). Sediment matrix. Note calcitic features in the form of limestone sand (l) and calcium carbonate concentrations (cc)

The top Unit IV section is up to 20 cm thick, relatively loose, with a continuous, light grey colour and with randomly embedded limestone gravel and cobble sized clasts. Underlying this light grey layer are shallow pockets up to 25 cm deep identified in the field as hearth-pit structures. These pockets are filled with dark grey/dark brown relatively loose sediment containing discrete concentrations of limestone cobbles (8–12 cm approx.). The lower boundaries of the hearth-pits are visibly affected by bioturbation. Brown-orange sediment corresponding to Units III and V marks the overlying and underlying sedimentary contacts of the deposit. The sediment of Unit IV is characterised by fine and medium sands consisting primarily of quartz, limestone and feldspar and chitonic — enaulic c/f related distribution (Fig. 4-a, b; see also Online Resource Table S1 for details of the micromorphological analysis and field data and Table S2 for textural characterization). A similar mineral composition can be observed in Units V and III, although an absence of calcitic sands was documented in the latter. Partial clay coatings are common in Unit IV — including in the hearth-pits — as well as in Units III and V. These features are mostly limited to the hollows on the quartz grain surface (Fig. 4c). Calcitic coatings are superimposed on these partial clay coatings in Unit IV — again also observed in the hearth-pits — and V, while almost none were found in Unit III (Fig. 4c, d, e). Unit III, interestingly, also lacks calcitic sands compared to Units V and IV.

The concentration of needle-fibre calcite in the dark grey/dark brown pockets of Unit IV is also remarkable (Fig. 4b).

Calcium carbonate values in Unit IV are consistent with micromorphological data showing a progression in its concentration from the upper contact with Unit III (5%) down to the lower boundary with Unit V (15%) (see Online Resource Table S2 for further details of soil chemistry). The pH values (between 8 and 9) indicate mild alkaline sediments in Units IV and V, but also in Unit III, despite evidence of mobilisation of carbonates down the profile. OM concentration presents a clear contrast between Units III and V (< 0.50) and the structures of Unit IV (up to 1,10) (see also the “Mesolithic Phase 2: SU 604, SU 625, SU 613 and SU 615” section). Iron-manganese orthic nodules and impregnations, together with local vughy porosity of the matrix in Unit IV and hearth-pits are frequent. These features are also observed in Units III and V to a lesser extent.

Finally, soil fauna activity and plant growth are commonly observed in Unit IV however, they are especially concentrated at two points of the NE and SE sectors (SU 604, AR-604–6 and SU 611, AR-611–3).

### Hearth-pit structures: field observations, micromorphology, texture and soil chemistry

All hearth-pits documented at the site were associated with Unit IV. The results of the analyses conducted on them are described according to the two established occupation phases.

#### Mesolithic Phase 1: SU 608 and SU 611

**Fig. 5:**
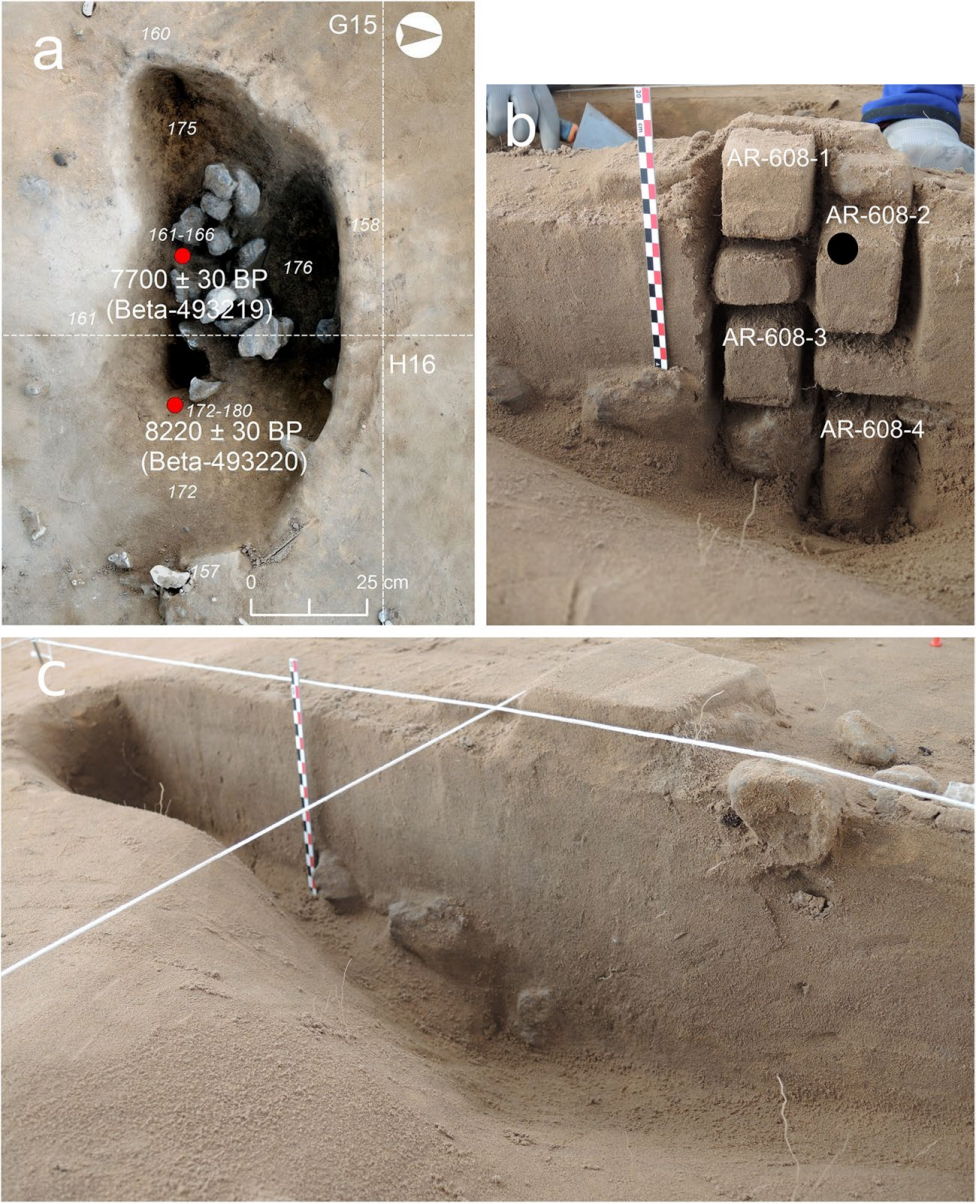
El Arenal. Mesolithic Phase 1 hearth-pit. **a** Ortho-photogrammetry of SU 608 from above. Note the burnt clast layering at the base of the structure. **b** Section of SU 608 showing the location of the block samples used for micromorphological analysis. The black point locates the OSL and TL sample analysed. **c** Section of SU 608. Note the burnt limestone layering between block sediment samples AR-608–3 and AR-608–4 at the base of the structure. AR-608–4 documents the contact of the structure with Unit V

Structure SU 608 presents a subrounded morphology and is relatively small (0.6 m long × 0.4 m wide × 0.2 m deep)**.** An accumulation of burnt carbonate rocks, with a relatively homogeneous size within the cobble range (10 cm approx.) and forming a layered pattern, was documented at its base (Fig. 5a)**.** Four micromorphological slides were studied to characterise the sedimentary infill (Fig. 5b, c). Its mineralogical composition is similar to that observed in the general sedimentary context of Unit IV. The microstructure is massive and presents a chitonic-enaulic related distribution and local vughy porosity. Inclusions of subangular calcitic coarse sands appear to be slightly more concentrated in the mid-lower section of the structure, in sample AR-608–3. Some of these calcitic coarse sands present the impact of fire (e.g., dark brown colour and dull lustre) and are associated with sand-silt-size charcoal fragments and rounded clayey aggregates of ash and charred OM. These combustion features in sample AR-608–3, were registered immediately above the burnt clast layering (Fig. 5c; Fig. 7a). The base of the structure in sample AR-608–4 also contains coarse calcitic sand inclusions; these, however, appear to be fresh, and charcoal remains are absent in the matrix of this section of the hearth-pit (Fig. 7b).

**Fig. 6:**
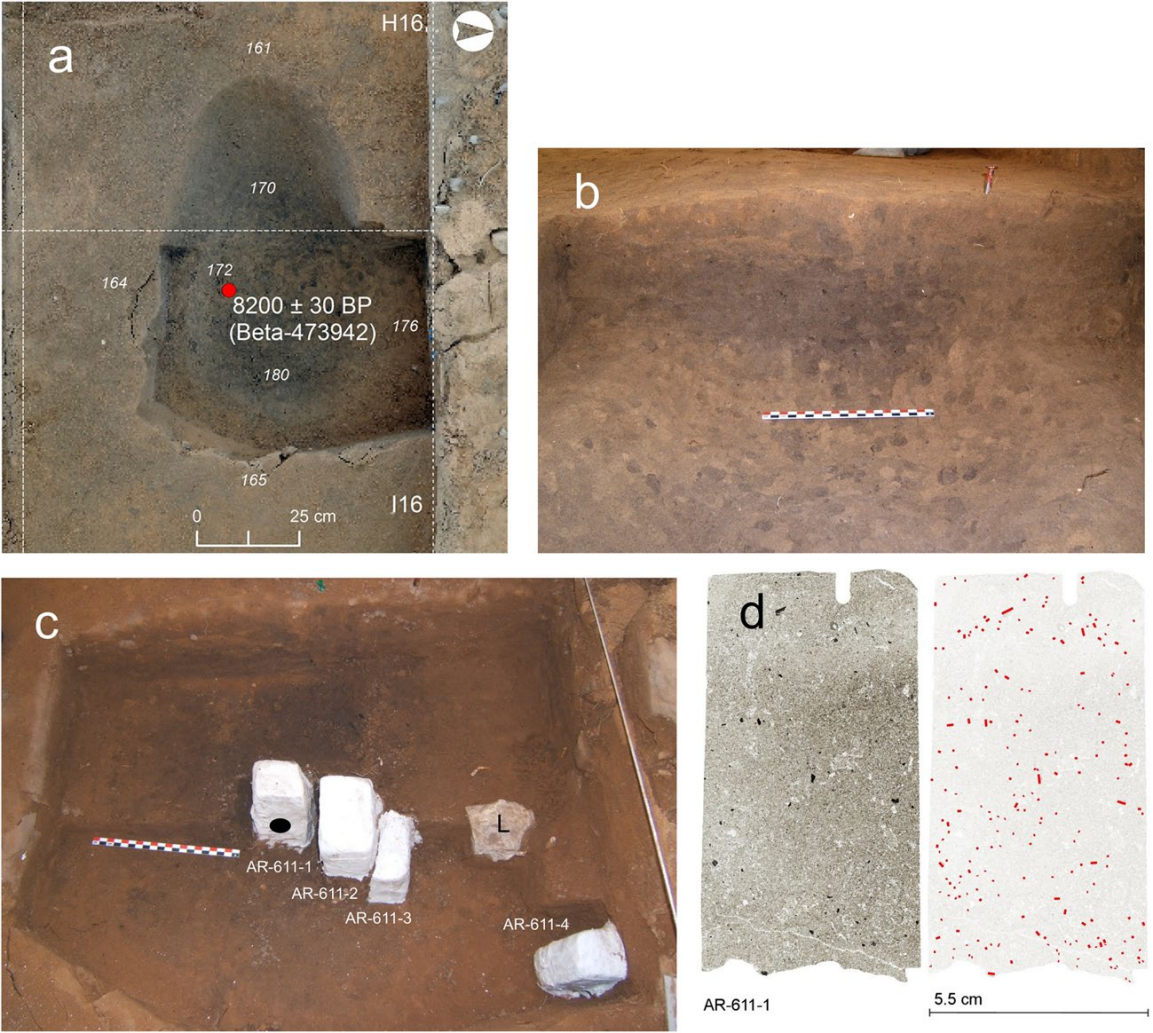
El Arenal. Mesolithic Phase 1 hearth-pit. **a** Ortho-photogrammetry of SU 611 from above. **b** Section of SU 611 in the field. Note the darker area from charcoal concentration at the centre of the structure. **c** Location of the block sediment samples used for the micromorphological analysis. Sample AR-611–3 documents one of the sedimentary discontinuities within Unit IV. Note location of sample AR-611–4 collected for reference outside of the structure. The black dot on block AR-611–1 indicates the location of the sample from SU 611 used for the OSL and TL analysis. Note the limestone accumulation (L) located outside the structure. **d** Left: scan of sample AR-611–1 micromorphological thin section. Visible dark particles correspond to charcoal fragments. Right: Distribution and orientation of sand-sized charcoals in the same sample

**Fig. 7:**
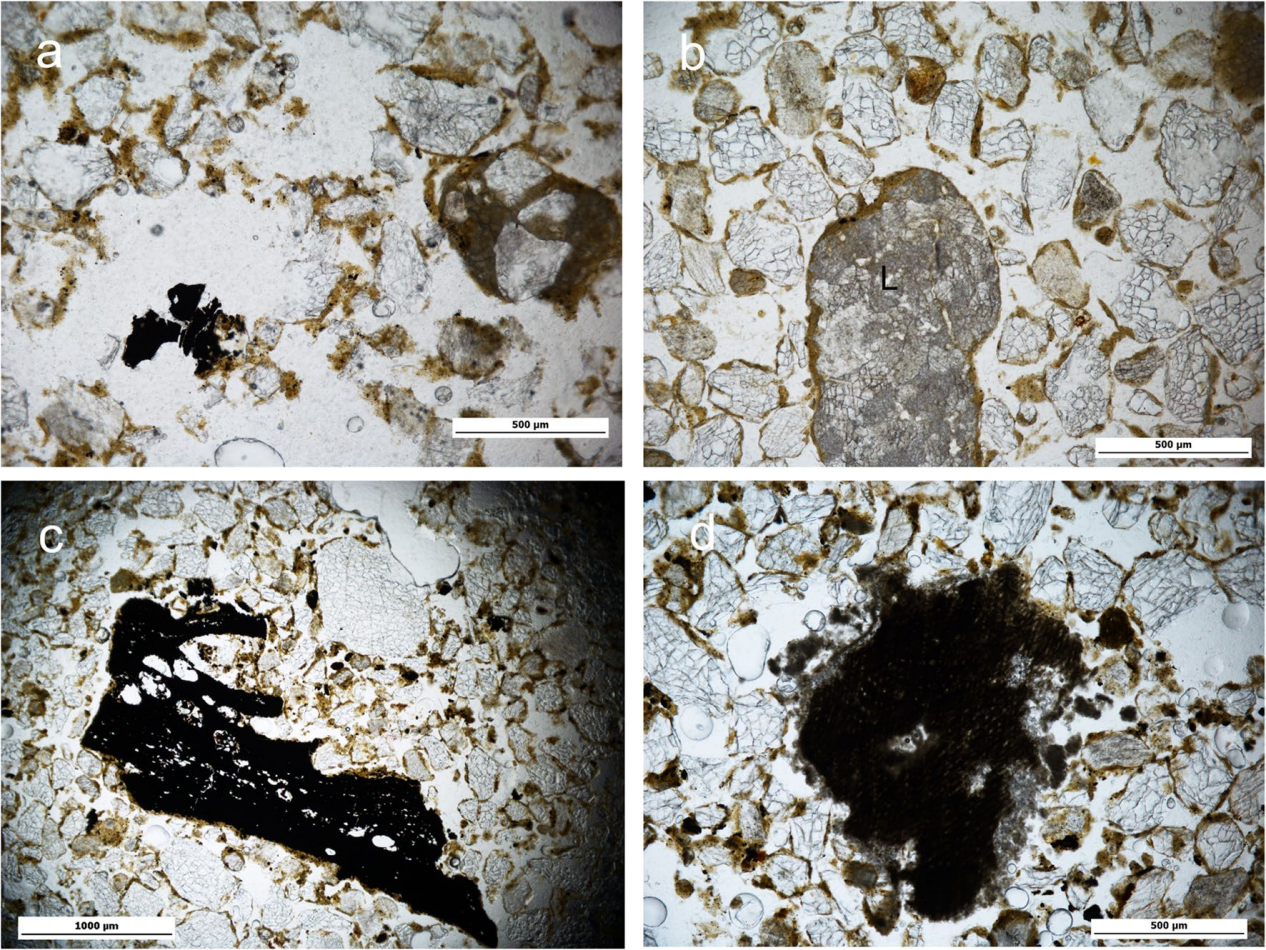
El Arenal. Mesolithic Phase 1 hearth-pits. Microscopic features. **a** Sample AR-608–3. Sand and silt-size charcoal concentration. PPL. **b** Sample AR-608–4. Lower contact of SU 608 with Unit V. Note lack of combustion residues and fresh limestone fragment. (L). PPL. **c** Sample AR-611. Charcoal fragment with traces of fire-induced changes in porosity. PPL. **d** Sample AR-611. Ash fragment. PPL

SU 611 is an oval structure with similar dimensions to those of SU 608 (0.6 m long × 0.4 m wide × 0.2 m deep) (Fig. 6a). There were no rock fragments inside the dark sediment of the structure, but they were recorded in a cluster located on the outer side of the hearth-pit. Three micromorphological thin sections document the vertical and lateral variability of the sediment infill. One additional slide from outside the structure provides reference data (Fig. 6b, c). SU 611 and SU 608 are similar in terms of microstructure, composition and matrix features, except for significantly larger and more abundant combustion residues in the earlier. Sand and silt-sized charcoal with opaque areas and loss of plant porosity and occasional bubbles from fire, together with ash pseudomorphs and infillings are common and randomly distributed (i.e., layering or local concentrations have not been observed) from top to bottom of the structure (Fig. 6d; Fig. 7c, d). A shift from chitonic to enaulic c/f related distribution between the central (AR-611–1 and AR-611–2) and the outer (AR-611–3 and AR-611–4) sections of the hearth-pit is observed, which is linked to the concentration of fine sediment (i.e., OM and fire residues) in the earlier. Bioturbation features (e.g., soil fauna excrements and skeletons, shell fragments, calcite biospheroids, root channels, plant tissue) in addition to acicular calcite are frequent. Subhorizontal and verticalised silica phytoliths (both loose and articulated, some of them charred) are randomly distributed.

The sediment is massive with the exception of a millimetric continuous, bioturbated layer detected in the outer section of the structure. Interestingly, this microfacies marks a stratigraphic discontinuity linked to a paleosurface within Unit IV.

#### Mesolithic Phase 2: SU 604, SU 625, SU 613 and SU 615

**Fig. 8:**
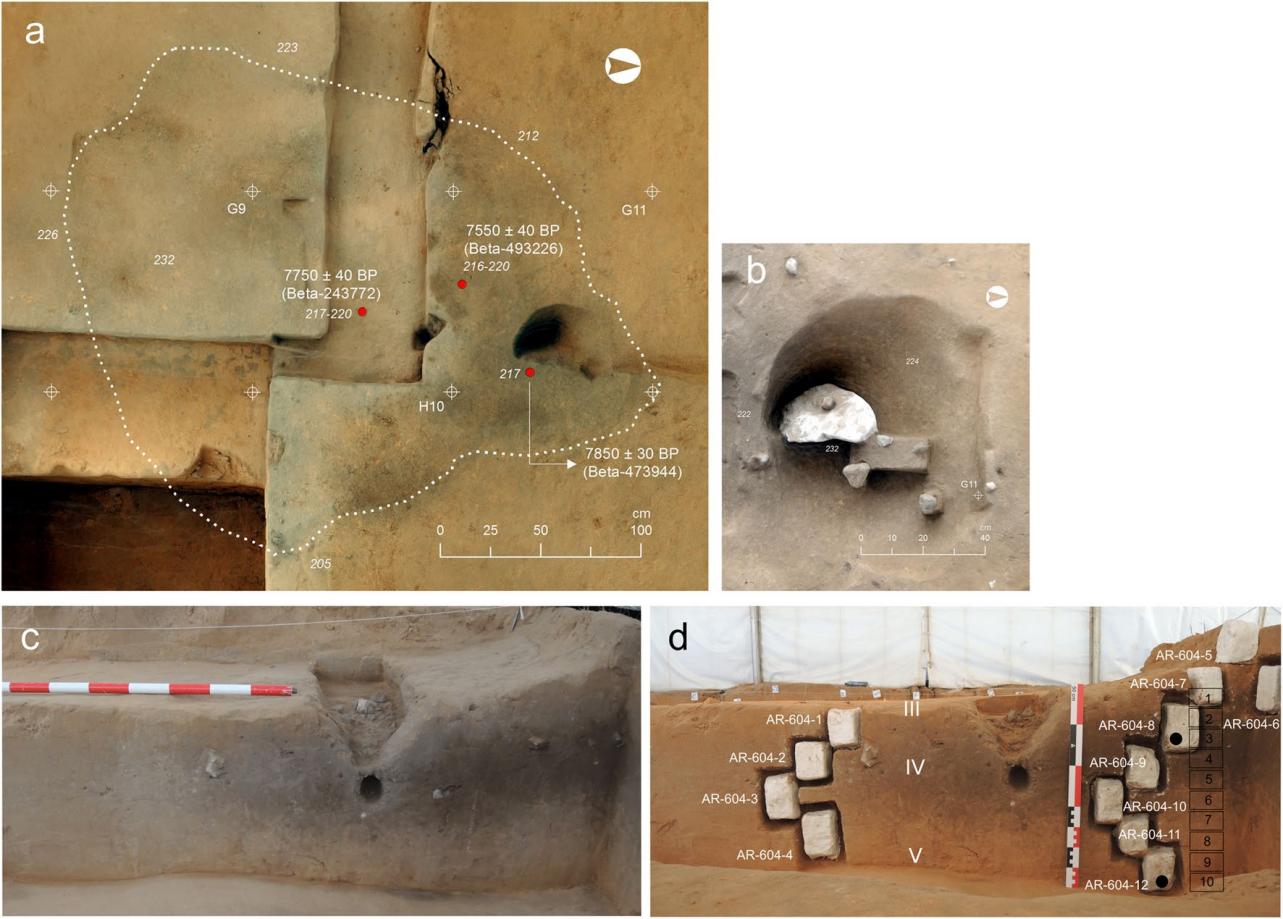
El Arenal. Mesolithic Phase 2 hearth-pit. **a** Ortho-photogrammetry view of SU 604 from above. The dotted line indicates the limits of structures SU 604 and SU 625. **b** SU 625. Note the vertical limestone boulder tilted at a 45° angle and location of the void left after removal within the limits of SU 604 in a) to the right of the image. **c** Section of SU 604 from the south. **d** Location and distribution of plastered block sediment samples in SU 604 used for the micromorphological analysis and corresponding sedimentary units. Black boxes (1–10) indicate the location of the bulk samples used for the soil chemistry and textural analysis. Black dot at the top indicates the location of OSL and TL sample in SU 604. Black dot at the bottom locates one of the reference samples for OSL and TL in Unit V

Results of SU 604 and SU 625 are presented here together because these structures are considered to be related in terms of functional usage**:** SU 604 presents a subrounded morphology and is considerably larger than the structures of Phase 1 (3 m long × 2.35 m wide × 0.25 m deep) covering a surface of 5 m^2^. Rock assemblage within is randomly distributed and show a greater size variability (i.e., from a pebble size to a boulder size) compared to those associated with SU 608 and SU 611. For its part, SU 625 is located in the northeastern corner of SU 604 and is much smaller (0.32 m long × 0.18 m wide × 0.25 m deep). It contains, however, a 0.40-m-long limestone block, thrust in at a 45° angle (Fig. 8a, b).

The SU 604 sedimentary infill is light grey in its upper section and dark grey-brown in the lower (Fig. 8c). Ten bulk samples for soil chemistry and texture and 12 sediment blocks for micromorphological analysis document the sedimentary infill of the feature and the stratigraphic contacts with the upper and lower units III and V (Fig. 8d). The micromorphology showed that the basic mineralogical composition is quartz and carbonate sands, with a predominance of fine and medium sands as observed in the structures of Phase 1. The microstructure is also similar to that of the structures of Phase 1: chitonic-enaulic c/f related distribution and locally vughy, with channels and planes. No microstratification was detected. Carbonate values were distinctly lower at the contact of the structure with the overlying Unit III (B-604–1: 5%), although significantly higher along the infill and at the base (peaking at 10% in samples B-604–5-10). This correlates with micromorphological observations of widespread calcitic coatings, nodules and concretions and a textural pattern transitioning locally from the sandy top section towards sandy loam. In contrast, the top section at the contact of Unit III largely lacks crystallitic b-fabric as well as calcitic sands and pedofeatures (Fig. 4c), suggesting that illuviation was involved in the formation of the calcitic features observed below.

**Fig. 9:**
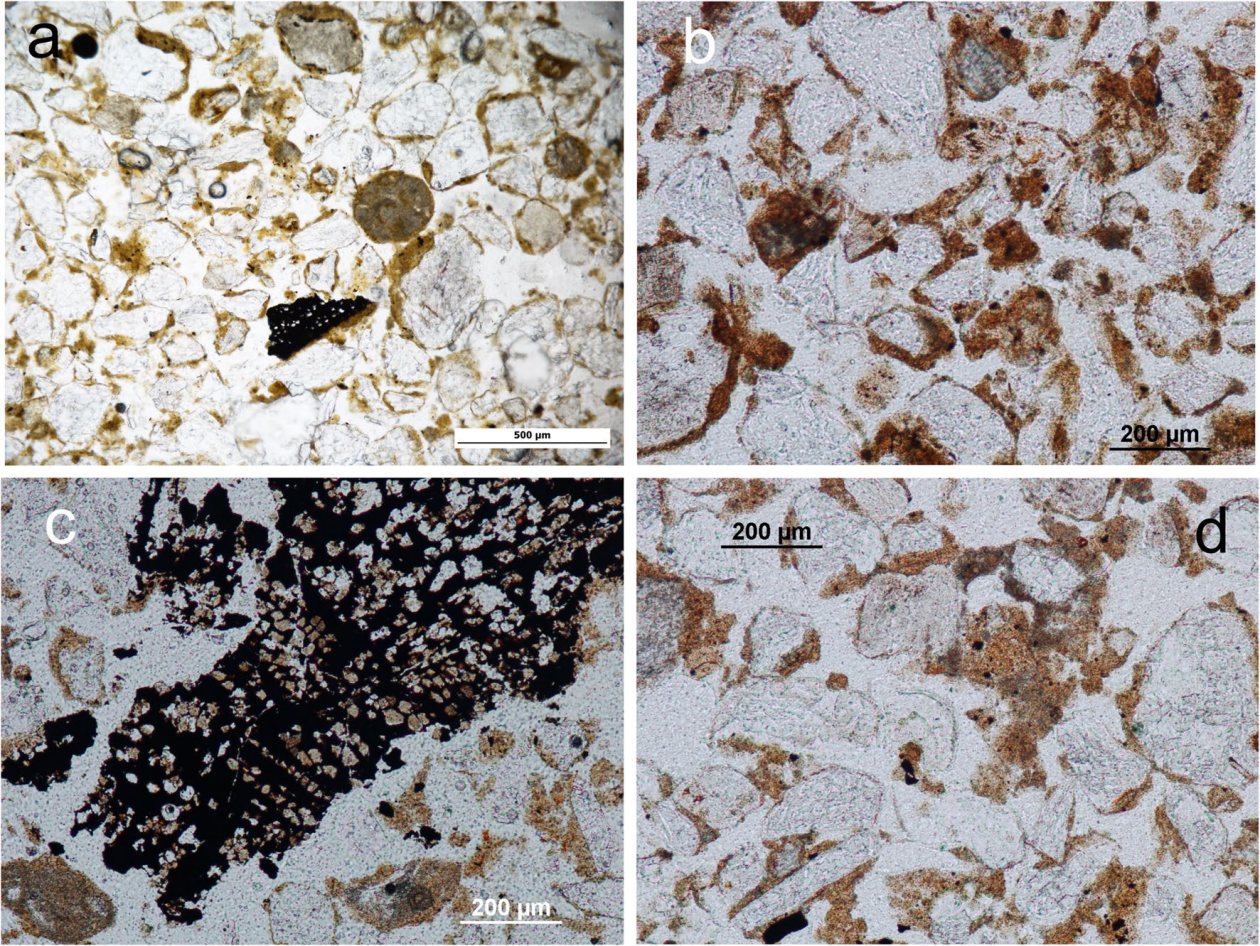
El Arenal. Mesolithic Phase 2 hearth-pits. **a** AR-604–5 bottom. Matrix general view of dark sediment in the SU 604 hearth-pit structure. Note sand and silt-size charcoal concentration. PPL. **b** AR-613–2-bottom. Matrix showing humus concentration in SU 613. PPL. **c** AR-613–1. Charcoal fragment. PPL. **d** AR-613–3 bottom. Calcitic features concentration. PPL

The OM content is relatively high and presents similar concentrations along the structure, with the maximum value detected in the darker sediment (B-604–3: 1.10) in contrast to the base of the structure at the contact of Unit V (B-604–10: < 0.50). Under the microscope reddish humus (i.e., coatings and intergranular patches), together with charcoal, makes up most of the OM, from top to bottom; the latter however is mainly associated with the darker section of the hearth-pit (Fig. 9a). Charcoal is most frequently represented by comminuted remains, although sand-size fragments are also observed, in addition to random ashed plant pseudomorphs. Charcoal (either sand or silt-sized grains) and humus are remarkably sparse from the contact with Unit V. Traces of soil fauna, plant growth and biological activity, such as enchytraeid excrements, plant roots sections and needle-fibre calcite, are frequent and particularly associated to the darker infill of the structure. Mn-Fe orthic nodules are abundant from top to bottom of the hearth-pit, which, together with local vughy porosity, indicates temporal water logging conditions. The contact between Units III and IV presents sharp boundaries with evidence of increased bioturbation.

Two bulk samples and one thin section from SU 625 were studied, focusing on grain size, soil chemistry and micromorphology. The mineral composition, textural pattern as well as the microstructure, porosity, b-fabric and pedofeatures related to calcium carbonate and Mn-Fe oxides are similar to those observed in SU 604. Carbonates present slightly lower values with respect to SU 604 (9–8%) while OM content is significantly lower (< 50–0.53%), which correlates with a much lower concentration of charcoal in thin section, still present, however, in the form of dispersed angular sand- and silt-sized inclusions.

SU 613 has also an irregular morphology and a larger size with respect to the structures of Phase 1 (1.17 m long × 1.08 m wide × 0.17 deep). Rock fragments are in the cobble size range and are randomly distributed from top to bottom as in SU 604, although some superpositions were observed in the field. Eighteen samples for soil chemistry and texture and six thin sections for micromorphology documented both vertical and lateral variability inside and outside the structure.

Again, the sedimentary infill is massive, with a lighter grey colour at the top in contrast to a darker base. The mineral composition and characteristics of the matrix are also similar to those observed in SU 604.

An OM increase was detected from top to bottom of SU 613 structure (B-613–0: < 50% vs. B-613–6 and B-613–1-top 0.81–0.82%), correlating with concentration of charcoal and reddish patches of humus observed under the microscope. The latter is dominant in the upper section and the outer area of the structure. As in SU 611, charcoal fragments present alteration of porosity from combustion. A progressive accumulation of carbonates was also observed towards the base of the structure (B-613–0: 3% — B-613–2: 15%) (Fig. 9d). Lower concentrations of OM (B-610–2: 0.57–0.63%), carbonates (B-610–2: 5–9%) and calcitic pedofeatures (e.g., calcitic nodules and impregnations) associated to the bulk and block samples located outside the structure mark the boundary with Unit III. Lower OM values are also associated with the contact with Unit V and the structure substrate (B-613–2-BASE: 0.65%).

The mid-bottom darker section of SU 613, where charcoal is concentrated, presents notable characteristics: the upper part (AR-613–1, AR-613–2-top) shows dispersed, angular, larger-size and well-preserved charcoal fragments, some of which present evidence of in situ breakage, probably from trampling. Random ash infills can be observed locally, in addition to bioturbation features and decayed OM (channels, soil fauna excrements, plant roots, reddish patches of humus) (Fig. 9b, c). Increased inputs of coarse sands and occasional gravel are concentrated in this section and are similar to the pattern observed in the sample located outside the feature at the contact of Unit III (AR-610–2), indicating the impact of surface exposure. In contrast, in the lower part (AR-613–2 bottom), where the matrix is more homogeneous, the bioturbation is lighter and comminuted charcoal is associated with small burnt bone fragments.

Finally, SU 615 presents a subcircular plan, similar dimensions to SU 613 (1.16 m long × 0.92 m wide × 0.25 m deep), a massive sediment infill, and dispersed random rock fragments. The results of the study of the two micromorphological thin sections from this feature show mineral content and textural values similar to those observed in the other hearth-pits from Phase 2, especially in SU 613. Sand and silt-sized scattered charcoal fragments were also observed.

### Carbonate rock assemblages

#### Experimental burning and petrography of reference carbonate rocks

The results allowed us to establish three main parameters of fire-induced changes on the rocks investigated, based on colour, development of oxide features, as well as porosity and fissure patterns (see Online Resource Table S3 for details on the reference material and experimental work).

**Fig. 10:**
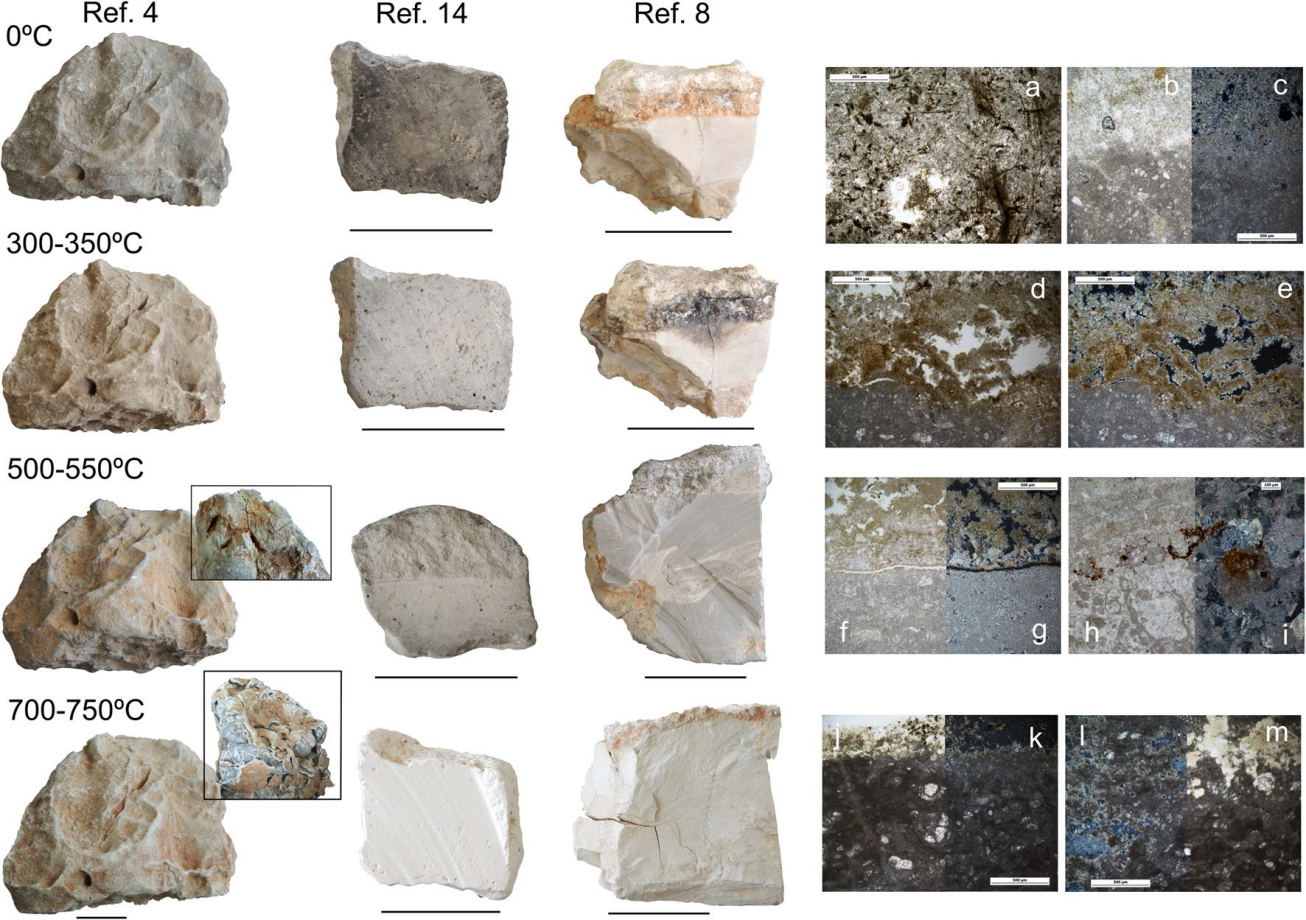
Reference and experimentally burned carbonate rocks. Left: macroscopic view of whole and sectioned specimens. The samples consist of bioclastic limestone (Ref. 4 and Ref. 8) and marl (Ref. 14) Lines from top to bottom: unburned specimens, samples burned at 300–350° C, 500–550° C and 700–750°. The inset of the specimen burned at 550–550 °C shows a detail of oxide concentration and fissure development from combustion. The inset of the specimen burned at 700–750 °C shows a detail of the effects of successive burning and exposure on the limestone sample. Note the colour variability and texture changes of the different samples due to burning. Right: microscopic views of sectioned samples. **a** Ref. 14. Unburned. Detail of fungal hyphae concentration. PPL. **b** and **c** Ref. 8. Unburned. Contact of weathered cortex (light grey top area) and bioclastic matrix (darker bottom area) in PPL and XPL. **d**, **e** Ref. 8. Contact of the weathered cortex and matrix burned at 300–350° C in PPL and XPL. **f**, **g** Ref. 8. Contact of the weathered cortex and matrix burned at 500–550° C in PPL and XPL respectively. **h**, **i** Ref. 8. Detail of oxide concentration from combustion at 500–550° C in PPL and XPL. **j**, **k** Ref.8. Contact of the weathered cortex and matrix burned at 700–750° C in PPL and XPL. **l**, **m** Ref.8. Detail of intense darkening, isotropy and increased porosity from calcination of the limestone matrix in XPL and PPL. Scale = 2 cm

Colour was the most obvious change at a macroscopic level (Fig. 10). Combustion at 300–350 °C entailed partial oxidation of the OM contained in the reference specimens and produced browning and blackening (on the limestone) and lightening (on the mudstone) of the original light beige and grey colours of the samples. At 550 °C, however, a grey tonality dominated in all samples, in addition to surface dullness. A shift towards a whitish tone was observed at 700 °C, paired with friability and a chalky texture.

Alterations in colour due to the combustion of the samples were also noticeable under the microscope. The porous, weathered, unburned cortex of the bioclastic limestone shifted from light grey to brown-reddish at 300–350 °C (Fig. 10b, d), to light brown-grey at 500–550 °C (Fig. 10f), and to dark grey-blackish at 700–750 °C (Fig. 10j, m) in PPL. The calcite cement showed also variability in colour with increasing temperature: the grey colour of the fresh bioclastic limestone darkened at 300–350 °C (Fig. 10c, e), shifted to a light reddish-brown at 500–550 °C (Fig. 10g), and finally turned very dark grey and dull at 700–750 °C (Fig. 10k, l). The mudstone sample, however, showed much less contrasting colour changes and only developed a light greyish brown shade at 700–750 °C, with no apparent loss of crystal lustre and pleochroism.

The development of oxide features (e.g., small nodules, coatings around calcite grains, and void and matrix impregnations) are evident both at macroscopic and microscopic levels at 500–550 °C (Fig. 10h, i), while they tend to disappear at 700–750 °C (Fig. 10j, k). It is interesting to note that higher concentrations of oxides were observed in bioclastic domains in contrast to micritic-sparitic matrices.

Unburned reference samples frequently presented variable degrees of physico-chemical alterations (e.g., porous and brittle cortex, rounded edges, fissures and occasional deep cracking) both at macroscopic and microscopic levels. Combustion did not seem to increase fissures between 300 and 350 °C, although brown and dark brown coatings started to develop occasionally in voids. However, the increasing of the temperature to 500–550 °C, multiplied and deepened the existing cracking, as visible to the naked eye and under the microscope (Fig. 10). This pattern intensified with combustion at 700–750 °C, especially in the bioclastic limestone, which also presented substantial cortex loss at this temperature (Fig. 10j. k,). Nevertheless, the specimens held together when the samples were allowed to gradually cool down inside the furnace. Interestingly, the sample burned at 500–550 °C (Ref 8) and immediately removed from the furnace after combustion suffered from partial collapse.

Intense cracking and fissuring of cortex and fragmentation were documented for the non-sectioned sample (Ref 4) following the completion of combustion events between 300 and 700–750 °C and exposure to room conditions. The latter finding builds on the conclusion that limestone taphonomy is determined by the interaction of burning and post-combustion environments.

Microscopic alteration features of the carbonate reference samples prior to burning consisted of cortex porosity as well as corrosion from growth of fungal hyphae (Fig. 10a). Regarding the latter features, experimental firing entailed a shift in colour, from grey-brown, with reddish shade in some cases, in unburned and charred at 300–350 °C samples, to dull grey at 700–750 °C.

All samples underwent weight loss from combustion, especially at 700–750 °C; interestingly Ref. 14 (relatively small sectioned micritic carbonate sample with visible growth of fungal hyphae) presents the highest value (18%), in contrast to Ref. 4, which yielded the lowest one (0.6%). These results (see also graphic in Online Resource Table S3) suggest that size and possibly cortex in addition to the OM and water content are relevant factors to be considered when assessing the impact of fire on limestone and carbonate rocks.

#### Petrography of carbonate rocks from the hearth-pits

**Fig. 11:**
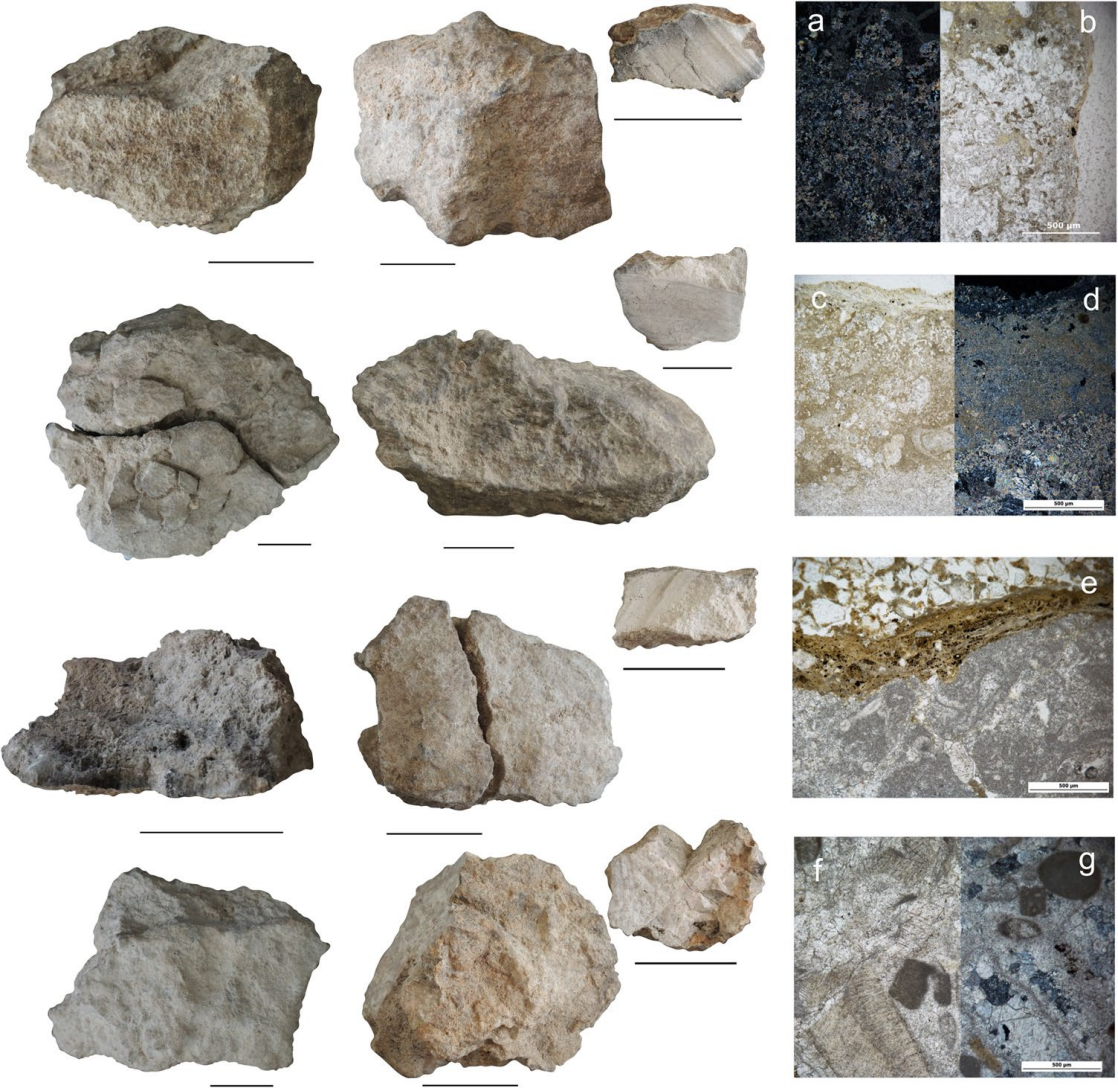
El Arenal. Archaeological carbonate rock samples from the hearth-pits studied. Left: macroscopic view of specimens and section cuts for petrographic thin section production (scale = 2 cm). From top to bottom: samples SU 604, SU 608, SU 613 and SU 611. The sample from SU 608 was collected from the rock layering at the bottom of the structure (Fig. 5b, c). Right: microscopic views of petrographic and micromorphological thin sections. **a**, **b** SU 604. Detail of the contact between the cortex and the inner matrix of the sectioned specimen on the left. PPL and XPL. **c**, **d**, SU 608. Detail of the contact between the cortex and the micritic matrix of the sectioned specimen. PPL and XPL. Note the light brown-grey colour of the cortex and small oxide concentrations similar to traits observed in experimental samples burned at 500–550 °C (Cfr. Fig. 10f, g). **e** SU 613. Partial view of a limestone fragment in micromorphological thin section showing compacted clayey ash coating with embedded silt-sized charcoal. PPL. **f**, **g**, SU 611. Detail of the fossil-supported matrix limestone. PPL and XPL

Macroscopically, Phases 1 and 2 rock assemblages frequently presented a grey and dark-grey colour with occasional black and red patches due to combustion, similar to those observed in the experimental samples. In addition, physico-chemical alterations of the cortex were common (i.e., a porous and brittle cortex with calcium carbonate concretions). Some specimens appeared also less dense than the parent material, and they showed deep cracks and in situ fragmentation (Fig. 11) in addition to a friable inner matrix.

Under the microscope, the SU 608 sample displayed a light brown-grey porous cortex with small oxide inclusions, while the micritic-sparitic matrix appeared light grey. Embedded fossils showed a reddish-brown shade and a significant darker grey colour, in agreement with features observed on our experimental samples of biosparite limestone burnt between 300–350 °C and 500–550 °C (Fig. 11c, d). Phosphatised ash coatings (light yellow-grey clayey coatings around the cortex with silt-size charcoal inclusions and isotropic in XPL) were also observed.

Darkening of fossils, slight brown-reddening of the matrix and slight oxide concentration were also recorded in the limestone sample from SU 611 (Fig. 11f, g).

Limestone from SU 604 presented also coatings consisting of phosphatised ash with embedded microcharcoal (Fig. 11a, b), owing to contact with burnt plant tissue. Other features observed include the darkening of calcitic fossils and pore walls to a brown-grey colour, and the dark grey colouring of fungal hyphae and fissures. This was compatible with the combustion traits of the experimental samples that had been burned at 500–550 °C and suggests burning at moderate-high temperature of the archaeological rocks. All these traits come from the dark hearth-pit infill documented both for the micromorphological (i.e., M 604–9 and M 604–10) and petrographic thin sections. SU 613 samples also presented ashy coatings, in addition to the darkening of the matrix and grey-brown coatings around pores and fissures. However, the general darkening of the matrix in SU 613 samples is less apparent than in SU 604 (Fig. 11e). Since samples from SU 613 are more finely grained, lower thermal impact on the matrix as showed by our experiments could explain this feature. In addition, recarbonation after combustion may have masked burning traits (Canti 2017).

Lithology of the reference calcitic rocks (Online Resource Table S3) presents similar characteristics to those associated to the hearth-pits.

### OSL and TL: heating of the hearth-pit sediments

**Fig. 12:**
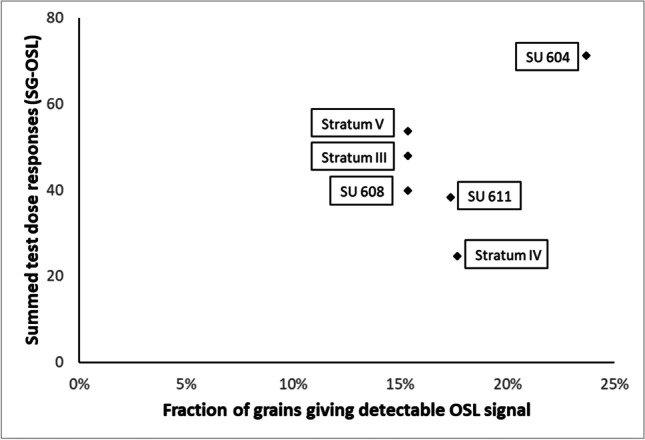
Single-grain OSL characteristics of the 6 studied samples: intensity of the fixed test dose signal (summed over all measured grains, given in arbitrary units) as a function of grains for which the uncertainty on the test dose signal is less than 15%

Results of the OSL and TL analyses of hearth-pit and reference samples showed that the fraction of grains that emitted a detectable signal ranged from 15 to 24%. While five of the tested samples yielded values between 15 and 18%, a total of 24% of the grains of the SU 604 hearth-pit sample (AR-OSL-604–8) (Fig. 12; Table 2) gave detectable light. The latter also produced the largest summed OSL signal value, whereas the other samples provided similar results — except sample AR-OSL-B-PF-4, which gave a much weaker OSL signal. No significant variation could be observed for the median OSL signal. Finally, once again, the TL emission was the most intense in the case of the SU 604 sample (AR-OSL-604–8).

**Table 2:** Summary of the TL and single-grain OSL measurements

Feature/unit	Sample ID	sTn < 15%	Sum Tn	Median (Tn)	Sum Max TL
SU 604	AR-OSL-604–8	0,24	71,259	235	7300
SU 608	AR-OSL-608–2	0,15	39,836	323	3950
SU 611	AR-OSL-611–1	0,17	38,293	234	4375
Unit IV	AR-OSL-PF-4	0,18	24,720	182	4250
Unit III	AR-OSL-PF-12	0,15	47,982	300	5150
Unit V	AR-OSL-604–12	0,15	53,655	245	3300

‘sTn < 15%’ denotes the fraction of grains for which the test dose signal uncertainty is less than 15%; ‘Sum Tn’ is the intensity of the test dose signal, summed over all measured grains; (in photon counts, so arbitrary units); ‘Median (Tn)’ denotes the median of all measured single grain test dose OSL signals (idem); ‘Sum Max TL’ is the maximum TL intensity of the 110 °C TL peak measured during the preheating prior to the dose measurement, summed over the 3 disks measured for each sample (idem).

To summarise, only the sample from the SU 604 hearth-pit (AR-OSL-604–8) showed a clear heating-induced change in luminescence characteristics, visible both in the fraction of grains emitting OSL, the summed OSL signal in response to a fixed dose, and the intensity of the 100 °C TL peak. According to Bøtter-Jensen (2000), the sensitivity increase requires heating temperatures above 400 °C. One may therefore deduce that the SU 604 hearth-pit sample (AR-OSL-604–8) was heated slightly above this temperature. Discussion of the values produced by the other tested hearth-pits is provided later in the text.

Despite our results are only a preliminary exploitation of luminescence characteristics for such issues; these outcomes indicate that luminescence indeed has the potential to characterise past heating. Our data also demonstrate that the absence of particular care taken during sampling does not prevent from useful results to be obtained, i.e., from observing heat-induced sensitivity changes. This is a significant practical advantage, as our sampling was quite straightforward; in particular, luminescence measurements did not require to be planned beforehand.

Several lines of investigation might be explored in the future: e.g., experimentally characterise the evolution of sensitivity as a function of temperature for the studied samples (this evolution is probably quite material-specific); evaluate the sample conditioning — using opaque bags to prevent UV exposure could improve the sensitivity of the method; or investigate feldspar infra-red stimulated luminescence as another proxy.

## Discussion

### Sediments and formation process of Unit IV

The textural pattern documented in Unit IV indicates that the substrate of the hearth-pits is made of coarse dust (Livingstone and Warren 2019) from local and short distant sources, which accumulation was favoured by poor soil and vegetation cover (Vandenbergehn 2013). Inputs of coarse fraction at the top of the deposit are evidence of disturbance by surface processes. Comparison of the lithology of the calcitic rocks associated to the hearth-pits against that of the reference specimens’ points to the nearby Sierra del Castellar outcrop as the source of this coarse debris. In addition, the chitonic related distribution of the sediments suggests a slow reworking of the deposit by surface dynamics (Mücher et al. 2010).

The frequent occurrence of iron-manganese orthic nodules and impregnations, together with the local vughy porosity of the matrix in Unit IV and hearth-pits are linked to short-term waterlogged conditions in the presence of organic matter. Such conditions probably originated in a water table rise, slope events, or flooding episodes affecting the lake margins where the site is located.

Clay coatings limited to the hollows on the quartz grain surface, frequently observed in Unit IV and the hearth-pits — as well as in Units III and V, reveal the residual character of these pedofeatures and that they were transported with the sand to the site; therefore, they cannot be related to environmental conditions during the formation of the deposit.

In contrast, calcitic coatings superimposed on these partial clay coatings, and observed in Units IV and V while almost missing from Unit III (Fig. 4c, d, e), formed in situ as a result of illuviation from the upper sediments, contributing to carbonate concentration in the lower section of the profile. The absence of calcitic sands in III compared to IV and V supports this hypothesis and shows the impact of CaCO_3_ redistribution on the sediments. The development of calcitic pedofeatures points to marked surface moisture fluctuations (Khormali et al. 2006) after sand accumulation. Moreover, pedogenesis of carbonates is favoured by soil chemistry related to biogenic activity (Lal and Kimble 2000) a process observed in Unit IV of El Arenal (Fig. 4a, b) which is linked to Holocene warmer temperatures in semi-arid environments of SE Iberia (Candy and Black 2009).

Concentration of biological features and organic matter (residual in Units III and V) also suggests a period of general surface stability during the formation of Unit IV, which agrees with favourable conditions for human occupation of the site. The presence of a buried soil in the local area of El Arenal whose chronology overlaps that of Phase 2 (Tallón-Armada et al 2014) provides further grounds for wet and mild conditions during the occupation of Unit IV. Although the sediments of Unit IV appear massive, intensification of soil fauna activity and plant growth detected locally at microscopic level attest nonetheless, to the existence of paleosurfaces associated to sediment accretion hiatuses documented at the contact of Units IV and III, and also within Unit IV at the top of the SU 611 hearth-pit of Mesolithic Phase 1; the latter supporting the identification of successive occupation events at the site.

### Combustion traits, formation and function of El Arenal hearth-pits

Combustion residues are a major feature of Mesolithic sites (Brochier and Livache 2003) whose preservation, however, depends on the post-depositional impact of natural and anthropogenic dynamics (Wattez 1992). El Arenal is an open-air deposit, subject to surface conditions on a sandy substrate. These attributes, in combination with the type, intensity and frequency of activities at the site, determined how the interrelation of physico-mechanical, chemical and biochemical processes affected the preservation of fire traits. Therefore, they influence our ability to detect them and discuss their functional meaning during each occupation phase.

#### Mesolithic Phase 1: SU 608 and SU 611

Despite the preservation issues inherent to hearth-pit deposits, Mesolithic Phase 1 provided combustion traits, sedimentary features and a chronology that allowed us to propose functional hypotheses for each structure studied. Impact of fire on limestone cobbles was observed in SU 608 at a macroscopic and microscopic level. In addition, the concentration of charcoal and small burnt limestone debris detected in the sediments immediately above the thermo-altered rock lining suggests that the hearth-pit lower portion preserved the remains of the active combustion area of the structure (Wattez 2000). Furthermore, according to the features documented and their correlation with archaeological evidence, SU 608 is interpreted as a possible oven structure, in accordance with one of the most common explanations proposed for hearth-pit functions (Groenendijk 2004) and ethnographic examples (Yellen 1977). This hypothesis is also supported by the parallelism between SU 608 and archaeological earth ovens described in the literature where the pit structure and the burnt rock lining are interpreted as a prepared surface and the associated heating elements respectively (Black and Thoms 2014).

The OSL and TL results for SU 608 showed relatively low values that could be regarded as irrelevant in terms of thermal impact on the sediment. This may be due to the location of the sample in the upper section of the structure. Indeed, it presumably contains higher inputs of natural unburned sediments since it is not directly related to the combustion area preserved at the bottom, comprising burnt limestones and charcoal.

A radiocarbon date on a *Quercus* sp. evergreen charcoal sample from the top of SU 608, where the sediment for OSL and TL analysis was collected, yielded 7700 ± 30 BP (8547–8411 cal BP) (Fernández-López de Pablo et al. 2023). This age falls within the chronological frame of the second phase, and it is significantly younger than the date obtained for the base of the structure: 8220 ± 30 BP (9394–9027 cal BP). This evidence supports our hypothesis that the upper section of SU 608 contained significant inputs of unburned sediments from exposure and surface reworking that impacted the results not only of the radiocarbon dating, but also those of the OSL and TL analysis.

In contrast to SU 608, SU 611 presents a much higher concentration, larger size and finer preservation of combustion residues. Charcoal is the most evident organic combustion residue preserved at the site, and in SU 611 in particular, with a significant concentration of sand and silt-size fragments — including a high density of *Quercus* sp. and *Quercus* sp. evergreen specimens, based on our archaeobotanical analysis — considering both the chrono-cultural context (Jacomet and Vandorpe 2022) and the geomorphological setting. The original concentration of charred remains was possibly lessened by ash-induced charcoal disintegration, although diagnostic traits of this process documented in other sites (i.e., leached charred material and limpid clay coatings) (Huisman et al. 2012) were not detected in El Arenal. Ash pseudomorphs and infilling were also observed in a relatively high concentration in SU 611. However, patterns of carbonate redistribution and recrystallisation that affected Unit IV, partially hindered the possibility of distinguishing between geogenic carbonates and ashes. Moreover, this dynamic was favoured by high pH (Mentzer 2012).

The mild alkaline pH of sediments in El Arenal (i.e., values between 8 and 9) also encouraged silica dissolution (Cabanes et al 2011; Cabanes and Shahack-Gross 2015) since silica phytoliths were notably absent from the deposit. Nevertheless, dispersed phytoliths, some of which were charred, were observed in SU 611. Fast burial following a punctual/short accumulation event could have favoured the preservation of these phytoliths. A relatively high accumulation rate and the presence of loose and articulated aggregates — in accordance with discarding practices (Devos et al. 2009) — supports the interpretation of SU 611 as a dumping *locus* of combustion residues.

Burning traces were also documented in the limestones associated with this structure; they appeared, however, randomly accumulated on the outer side of the dark infill, thereby also enabling to link their accumulation to the disposal of combustion residues. The lower combustion residue concentration in SU 608 is compatible with the removal and disposal of charcoal and ashes elsewhere, regardless of the impact of surface processes on the top section of the structure at a later time. SU 611 is the most suitable location for such a dumping, based on the degree of preservation of the combustion residues, the statistically identical radiocarbon age, and the proximity to SU 608.

The OSL and TL analyses also provided a relatively low signal in the case of SU 611. This could be explained by the actions inherent to the cleaning of a combustion area. The ash and charcoal can be expected to have been removed by sweeping, but not the underlying hardened sediment, which implies that the sediment associated with combustion residues preserved in SU 611 may not correspond to the original burned substrate.

The presence of a sedimentary discontinuity in Unit IV associated with a paleosurface at the top of SU 611 could indicate an abandonment of the site. This is compatible with the chronological data, which indicates a temporal gap between Mesolithic Phases 1 and 2.

#### Mesolithic Phase 2: SU 604, SU 625, SU 613, SU 615

It has been shown that the substrate of Unit IV consisted primarily of sand deposits. These sediments were subsequently subject to pedogenesis favoured by heightened vegetation cover, moisture and temperature, suggesting ameliorated paleoenvironmental conditions with respect to Units V and III. In this scenario human activities and soil dynamics seem to have interacted in the formation of the hearth-pits of Phase 2.

A relatively high concentration of OM including charcoal and decayed plant remains, together with ash and burnt limestone in addition to biological activity detected in SU 604 is consistent with this hypothesis. Bioturbation favoured homogenisation of sediments and soil formation via a subaerial exposure of combustion residues mixed with further organic litter from the surface cover. We cannot rule out that this soil horizon originally extended beyond the SU 604 area. Indeed, the impact of aeolian and surface dynamics associated with the formation of Unit III may have affected its preservation outside the structure.

Post-depositional physico-chemical alteration of OM (i.e., humification) mingled with combustion traits, highlighting the complexity of this hearth-pit formation and the challenges inherent to their interpretation.

The OSL and TL values, together with combustion traits observed in the rock assemblage, attested to the heating of the SU 604 sediments well above the charring threshold. Furthermore, the presence of a rubified layer at the bottom of this feature, which had been documented in 2007 (Fernández-López de Pablo et al. 2011a), substantiates the hypothesis of in situ combustion (Röpke and Dietl 2017; Fierro-Vázquez et al. 2021).

In addition, experimental and archaeological works based on flat combustion features support the use of burnt artefact distributions to identify Mesolithic hearths (Sergant et al. 2006). Thus, the association of SU 604 with thermo-altered lithic refits and the spatial patterns of the artefacts (Rabuñal 2021; Fernández-López de Pablo et al. 2023) are compatible with a fire of anthropogenic origin related to the structure.

The size and shape of the structure further assists this affirmation: with a surface of 5m^2^, SU 604 is significantly bigger than the rest of the hearth-pits analysed here and some of the structures published in the literature (Peeters and Niekus 2017). Interestingly, its characteristics resemble some examples of Mesolithic dwelling structures documented in NW Europe (Niekus et al 2020), an interpretation reinforced by the multicomponent character of SU 604, where SU 625 would play the role of a posthole with a limestone wedge functionally related to a common activity area (Fernández-López de Pablo et al. 2023).

The relatively large and irregular morphology in addition to the weathering degree of the sediments of SU 604 adds a further dimension to our interpretation of the formation and function of the structure, which could be attributed to overlapping pit-structures, as reported in other archaeological sites (Groenendijk 1987; Verlinde and Newell 2006). Moreover, highly fragmented charcoal — possibly due to trampling (Huisman and Tebbens 2021), degraded combustion residues, humified OM, bioturbation and distribution patterns of the burnt coarse fraction indicate an in situ reworking and homogenisation of the sediments. Altogether this evidence suggests that SU 604 is a palimpsest — as opposed to an earlier hypothesis proposed (Fernández-López de Pablo et al. 2011a) — according to which the original configuration of the activity area was altered as a result of the combined impact of successive occupation episodes and soil formation.

In contrast to SU 604, SU 613 presents a more discrete and clearer spatio-temporal resolution of combustion residues at a microscopic level, despite the fact of also presenting homogenisation and soil dynamics. Increased bioturbation and detrital input in the upper section, in addition to some charcoal grains, fragmented in situ, attest to the occurrence of surface exposure and trampling, postdating the residue accumulation. The presence of burnt bone at the bottom of the structure may indicate an occupation surface that predates the combustion event (Mallol et al. 2013).

The irregular morphology and the large size of this hearth-pit mean that SU 613 could plausibly have been a hearth-related activity area. The spatial distribution of lithic artefacts increases the likelihood of this assumption: it clearly displays a statistically significant high cluster of thermo-altered specimens and lithic refits documented within the sediment infill. This was also the case for SU 615. Its location next to SU 613, added to the rest of its features, leads one to believe that it was part of the same activity area. Therefore, SU 613 and SU 615 — as well as SU 604 — could be the result of the palimpsest effect and the superposition of different structures that were possibly recurrently used. The archaeobotanical data showed higher densities of charcoal by volume (again, most of them corresponding to *Quercus* sp.), and the presence of pinecone scales that were radiocarbon dated (Fernández-López de Pablo et al. 2023). These two findings build on our hypothesis according to which SU 613 contains the remains of a combustion structure in situ, even though one affected by sediment homogenisation and soil dynamics.

A last question to be discussed regarding the combustion and function of the El Arenal hearth-pits is the evidence of opaque charcoal with altered porosity. This alteration feature, referred to by some authors as wood tar (Huisman et al 2019), derives from the fusion of plant structure due to combustion (Théry-Parisot 2001), and was recorded in charcoals from SU 611 and SU 613, and to a lesser extent from SU 604.

Experiments have linked this combustion residue, also identified as vitrified charcoal, with the burning of resinous wood (Py and Ancel 2006). However, the charcoal from El Arenal largely comes from *Quercus* species (Fernández-Lopez de Pablo et al. 2023). This prevents us from establishing a correlation between these alteration features and the intentional selection of wood type (e.g., based on burning properties) to explain their presence in El Arenal. In addition, the current data available indicates that the vitrification could not be related to combustion temperatures (Braadbaart and Poole 2008; McParland et al. 2010), hindering further its potential as a proxy to explore pyrotechnology at the site.

In the absence of further evidence, we currently believe that this trait could be the casual result of the particular firing environment (i.e., low oxygen/reducing conditions) (Théry-Parisot 2001) favoured by the hearth-pit morphology rather than an intended combustion by-product.

Some authors, however, have associated vitrification to taphonomic issues (Badal and Carrión-Marco 2004; Martínez et al. 2005), which needs also to be considered in the discussion.

As a final observation, hearth-pit functions should be interpreted within the wider context of subsistence strategies and the Mesolithic groups’ adaptation to Holocene environments (Groenendijk 1987). The biodiversity and readily accessible water supply provided by the Villena Lagoon ecological setting are likely to have played a relevant role in the occupation of El Arenal and the activities conducted at the site during the time frame corresponding to Unit IV. However, the complex formation of the deposit and its degree of preservation make it challenging to further detail the functionality of the hearth-pits, at least for the time being.

### Hearth-pits and intra-site occupation patterns

The investigation of the Mesolithic structures of El Arenal exemplifies how recognising and interpreting the so-called hearth-pits are not straightforward tasks. The term embraces a range of features with combustion traces affected by diverse post-depositional processes. To our mind, current debates on the origin of hearth-pits should not overlook the multiproxy information provided by the specific archaeological context of the structures. Equally, regional hearth-pit investigations aimed at inferring mobility as well as seasonality patterns (e.g., Niekus 2022; Müller et al. 2022) could benefit from this approach.

**Fig. 13:**
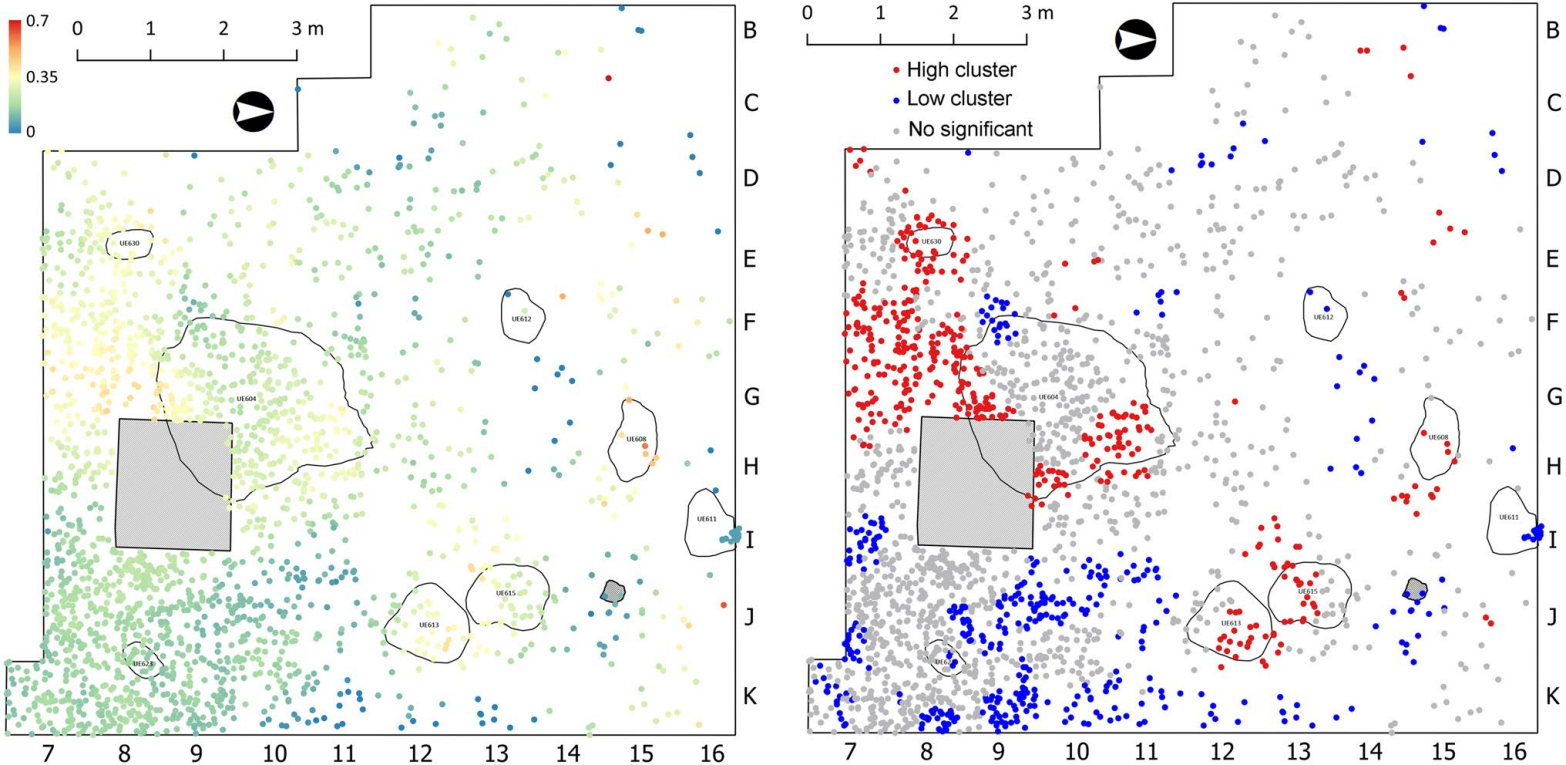
Spatial distribution of lithic assemblages regarding Unit IV occupation features. Left: Thermal alteration index; Right: Cluster analysis based on the Local Moran’s spatial autocorrelation index showing statistically higher and lower concentrations of thermo-altered lithic material (Rabuñal 2021). The shaded square represents the test trench excavated in 2006

With respect to El Arenal, the integration of the sedimentary data presented above and the artefact assemblage information regarding settlement evidence for each Mesolithic phase favour the interpretation of the anthropogenic origin of the El Arenal hearth-pits. The spatial distribution of thermo-altered lithic artefacts within Unit IV provides an independent proxy to assess the results of our geoarchaeological investigation of combustion facies. Figure 13 shows the presence of statistically significant high clusters of burnt lithics within the sedimentary infills of SU 608, SU 613, SU 615 and SU 604. This observation is consistent with the evidence provided by the micromorphology, the petrology of reference calcitic rocks, the soil chemistry and the OSL and TL analyses, indicating combustion in the structures. For SU 608, SU 613 and SU 615, we also found that the burnt lithic concentrations spread to the immediate adjacent areas of the hearth-pit limits, within a 1-m buffer zone. This in turn supports the primary and semi-primary nature of the sediments and, by extension, of the combustion areas serving as a substrate for the discrete spatial patterning of burnt artefacts.

Regarding the earliest Mesolithic phase (9.3–9.1 cal ka BP), features SU 608 and SU 611, interpreted as a possible oven and dumping structure, respectively, were associated to a relatively small and well-preserved area of the site, as suggested by assemblage integrity and confirmed by the presence of short distance refits (Rabuñal 2021; Fernández-López de Pablo et al. 2023). In addition, radiocarbon dates yielded by SU 608 and SU 611 were exactly similar and supported the proposed functional complementarity of the structures over the same occupation episode.

In contrast, for the second Mesolithic phase (8.7–8.4 cal ka BP), SU 604 and SU 613 reflected more intensively the combined impact of anthropic and natural post-depositional disturbance and alteration, including trampling, surface exposure and soil formation. This entailed the poor preservation of occupation surfaces, favoured by the homogenisation of sediments containing residues accumulated over the repeated use of activity areas.

Microstratigraphic evidence of fire, together with spatial distribution analyses of burnt lithics from these structures have shown how formation processes during and after occupation can affect the spatio-temporal resolution of the sedimentary record. This results in a palimpsest effect which impedes the discerning of individual episodes (Wojtczak and Ismail-Meyer 2017; Machado et al. 2013).

According to the Bayesian chronological models built on short-lived samples, Phase 2 occupation lasted 218 to 397 years (95.4% CI) in model 1, and 0 to 180 years (95.4% CI) in model 2 (Fernández-López de Pablo et al. 2023). In both scenarios, different occupation episodes may have occurred, probably spanning several generations. They left room for post-depositional disturbances triggered by human reuse of the space and possible combustion areas. Detailed spatial analyses of artefact assemblages of Unit IV statistically demonstrated a spatial association between lithic remains and combustion features (Rabuñal 2021), as well as the preservation of the original site structure. The identification by micromorphological analysis of an occupation surface at the base of SU 613 supports this interpretation.

## Conclusions

This work presents the results of the first multiproxy geoarchaeological and experimental investigation of Mesolithic hearth-pits discussed in their archaeological context.

By exploring the formation of the structures documented at El Arenal, we addressed one of the most common and debated Early Holocene features in Europe. Micromorphology, texture and soil chemistry, as well as newly introduced OSL and TL analyses allowed us to clarify the composition, microstratigraphy and formation of the hearth-pits in their sedimentary setting. Lithological study of the hearth-pit calcitic rocks revealed the source of the assemblage. Through the controlled burning of reference limestone and comparisons with archaeological data, we explored the value of experimental work to assess the impact of fire on hearth-pit sediments. Combustion residues were characterised and the effects of post-depositional and soil dynamics on fire remains were discussed. These outcomes allowed us to emphasise the role of human–environment interactions in the formation and preservation of open-air deposit occupation areas and their implications on archaeological interpretations.

The sedimentary data and the distribution of burnt lithic assemblages suggest that the origin of the hearth-pits documented at El Arenal was anthropogenic. In the light of the radiocarbon dates available, the investigated features were formed during two distinct and successive occupation phases within an overall short period of a few hundred years. The Mesolithic settlement and the formation of the hearth-pits took place in a context of sedimentary stability and subsidence, with relatively denser vegetation cover, higher moisture and warmer temperatures with respect to the more arid Pleistocene and Middle Holocene environments, pre- and post-dating the human occupation.

The variability of the morphology, dimensions and sedimentary traits documented for the two settlement intervals supports the hypothesis of differentiated usages and functions in each. Hearth-pits corresponding to the earliest phase were interpreted as possible oven and dumping structures used during brief/ephemeral occupation. The structures of the later phase were considered to reflect palimpsests of more structured activity areas formed over longer-term/recurring dwelling episodes involving fire events.

Our work provides a methodological baseline to determine the disparity of hearth-pit features using an integrated high-resolution approach, especially focused on the identification of burnt sediments and site formation process; this can be useful in larger scale analyses exploring occupation strategies.

We hope that our results contribute to a better understanding of the complex formation processes underlying the concepts of ‘hearth-pits’ and ‘Mesolithic dwellings’ when interpreting Early Holocene settlements. We also wish to highlight the need to critically evaluate the sedimentary and artefactual evidence associated with them in the light of geoarchaeological data.

## Supplementary information

Below is the link to the electronic supplementary material.

Supplementary file1 (DOCX 26 KB)

Supplementary file2 (DOC 95 KB)

Supplementary file3 (DOC 69 KB)

## Data Availability

All data are availability in the main text and the supplementary information of the manuscript.
