# Supplementary material for: Mesolithic hearth-pits and formation processes: a geoarchaeological investigation of sediments from El Arenal de la Virgen site (SE Iberia)
Source: Archaeol Anthropol Sci. 2023 Jun 22;15(7):104. doi: 10.1007/s12520-023-01794-5 (PMC10287818; doi:10.1007/s12520-023-01794-5)
Supplement: Supplementary file 1 — Supplementary file1 (DOCX 26 KB) [file 12520_2023_1794_MOESM1_ESM.docx]

| UNIT-HEARTH-PIT ID-MUNSELL SOIL COLOUR CODE | **FIELD DESCRIPTIONS** | **MICROSCOPIC FEATURES** |
| --- | --- | --- |
| **UNIT IV-GENERAL** | | |
|  | Top section= ≤ 20 cm thick; continuous; Greyish colour: 7.5.YR 6/4 (dry)-7.5.YR 5/4 (wet); relatively loose; random limestone gravels and cobbles (≤ 10 cm). Locally underlain locally by shallow pockets ≤ 20 cm deep (hearth-pits);  Lower and upper contact with brown orange sands from Units V and III: 7.5.YR 6/6 (dry) – 7.5.YR 5/8 (wet) / 7.5.YR 6/8 (dry) – 7.5.YR 5/8 (wet); lower boundary gradual from bioturbation linked to the base hearth-pit base; upper boundary with Unit III clear and locally erosive; | Fine and medium sands (subangular quartz, subrounded/rounded micritic and bioclastic limestone and feldspar; zircon and tourmaline inclusions); some mineral grains present ferruginous coatings (inherited feature).  Massive structure; c/f-related distribution: chitonic-enaulic; local vughy porosity; crystallitic b-fabric; partial clay coatings in the cavities of the sand grains (inherited);  OM: sand and silt-size charcoal; plant root remains; fungal spores; reddish patches (mineralized OM);  Pedofeatures: calcitic coatings around mineral grains; calcitic impregations of the fine matter; calcitic nodules and concretions; needle-shape calcite; iron-manganese orthic nodules and impregnations;  Paleosurfaces detected at the contact of Units IV and III (AR-604-6) and within Unit IV (AR-611-3); |
| **SU 608** | | |
| UNIT IV  AR-608-1  Dry: 7.5.YR 5/4; Wet: 7.5.YR 4/4  AR-608-2  Dry: 7.5.YR 5/2-5/4; Wet: 7.5.YR 4/2-4/4  AR-608-3  Dry: 7.5.YR 5/4; Wet: 7.5.YR 4/4 | Subrounded morphology (0.6m long x 0.4m wide x 0.2m deep)**; b**urnt carbonate rocks (layered pattern + relatively homogeneous clast size of about 10 cm) at its base; | Fine and medium sands (subangular quartz, subrounded/rounded micritic and bioclastic limestone and feldspar; zircon and tourmaline inclusions); some mineral grains present ferruginous coatings (inherited feature);  Massive structure; c/f-related distribution: chitonic -enaulic; local vughy porosity; crystallitic b-fabric; partial clay coatings in the cavities of the sand grains (inherited);  OM: sand and silt-size charcoal; red patches mineralized OM.  Pedofeatures: calcitic coatings around mineral grains; calcitic impregations of the fine matter; calcitic nodules and concretions; needle-shape calcite. iron-manganese orthic nodules and impregnations;  Combustion traits on calcitic coarse sands (e.g., dark brown colour and dull lustre); sand-silt-size charcoal fragments and rounded clayey aggregates of ash and charred OM; increased concentration of burning features in AR-608-3; |
| UNIT V  AR-608-4  Dry: 7.5.YR 5/2-5/4; Wet: 7.5.YR 4/2-4/4 |  | Same features as in samples AR-608-1; AR-608-2; AR-608-3; however, coarse calcitic sands lack combustion traits and charcoal remains are absent; |
| **SU 611** | | |
| UNIT IV  AR-611-1  Dry: 7.5 YR 5/2; Wet: 7.5 YR 4/2  AR-611-2  Dry: 7.5 YR 6/2; Wet: 7.5 YR 5/2  AR-611-3-top  Dry: 7.5 YR 5/2; Wet.5 YR 4/2  AR-611-3-bottom  Dry: 7.5 YR 6/2-6/4; Wet: 7.5 YR 5/2-5/4  AR-611-4 next to the hearth-pit structure)  Dry: 7.5 YR 6/4; Wet: 7.5 YR 5/4 | Oval structure (0.6 m long x 0.4 m wide x 0.2 m deep); lack of rock fragments in the sediment infill, but concentrated on the outer side of the hearth-pit (≤ 20 cm approx.); | Fine and medium sands (subangular quartz, subrounded/rounded micritic and bioclastic limestone and feldspar; zircon and tourmaline inclusions); some mineral grains present ferruginous coatings (inherited feature); ash pseudomorphs grains; soil fauna skeletons; shell fragments; articulated aggregates of silica phytoliths;  Massive structure; the central area of the structure (AR-611-1 and AR-611-2) combines vughy microstructure with chitonic - enaulic c/f related distributions, while in the outer portion of the hearth-pit (AR-611-3 and AR-611-4) enaulic c/f related distribution is dominant with decreasing concentration of fine matter towards the outer area of the hearth-pit; crystallitic b-fabric; partial clay coatings in the cavities of the sand grains (inherited);  OM: relatively high concentration sand and silt-sized charcoal, with partial vitrification, root channels, plant tissue;  Pedofeatures: calcitic nodules and infillings; needle-shape/acicular calcite; calcite biospheroids;  Millimetric continuous, bioturbated layer detected in the outer section of the structure (AR-611-3); |
| **SU 604** | | |
| UNIT III  AR-604-1  Dry: 7.5.YR 5/4; Wet: 7.5.YR 4/4  UNIT III / IV  AR-604-5  Dry: 7.5.YR 6/4; Wet: 7.5.YR 5/4  AR-604-6  Dry: 7.5.YR 5/4; Wet: 7.5.YR 4/4 -  Dry: 7.5.YR 6/4; Wet: 7.5.YR 5/4 | Subrounded 1 (3 m long x 2.35m wide X 0.25m deep) covering a surface of 5 m^2^; rock assemblage randomly distributed and size variability (pebble - boulder size); | Fine and medium sands (subangular quartz) and feldspar; zircon and tourmaline inclusions); some mineral grains present ferruginous coatings (inherited feature); lack of calcitic sands;  Massive microstructure; AR-601= c/f related distribution coarse monic – chitonic; speckle b-fabric; AR-605 and AR-606= c/f related distribution coarse monic – chitonic; speckle b-fabric at the top of the samples. AR-605 and AR-606= c/f related distribution chitonic-enaulic and crystallistic b-fabric at the lower portion of the samples;  Lack of calcitic pedofeatures, mineralized OM, plant tissue and biogenic activity traits;  Samples AR-605 and AR-604-6 contains random calcitic sands, sand-sized charcoal and ash pseudomorphs grains while largely lacking silt-size OM particles at the top portion; |
| UNIT IV  AR-604-2  Dry: 7.5.YR 6/4; Wet: 7.5.YR 5/4  AR-604-3  Dry: 7.5.YR 6/4; Wet: 7.5.YR 5/4  AR-604-4  Dry: 7.5.YR 6/4-6/6; Wet: 7.5.YR 5/4-5/6  AR-604-7  Dry: 7.5.YR 6/4; Wet: 7.5.YR 5/4  AR-604-8  Dry: 7.5.YR 5/2-5/4; Wet: 7.5.YR 4/2-4/4  AR-604-9  Top: Dry: 7.5.YR 5/2-5/4; Wet: 7.5.YR 4/2-4/4  Bottom: Dry: 7.5.YR 6/4; Wet: 7.5.YR 5/4  AR-604-10  Dry: 7.5.YR 6/4-6/6; Wet: 7.5.YR 5/4-5/6 |  | Fine and medium sands (subangular quartz, subrounded/rounded micritic and bioclastic limestone and feldspar; ash pseudomorphs, zircon and tourmaline inclusions); some mineral grains present ferruginous coatings (inherited feature);  Massive microstructure; c/f related distribution chitonic-enaulic locally vughy, with channels and planes; crystallistic b-fabric;  OM: reddish patches (mineralized); charcoal (sand-silt sized); plant tissue and root sections;  Pedofeatures: calcitic coatings, nodules and concretions; acicular calcite; enchytraeid excrements; Mn-Fe orthic nodules; |
| UNIT V  AR-604-11  Dry: 7.5.YR 6/4-6/6; Wet: 7.5.YR 5/4-5/6  AR-604-12  Top: Dry: 7.5.YR 6/4-6/6; Wet: 7.5.YR 5/4-5/6  Bottom: Dry: 7.5.YR 7/6; Wet: 7.5.YR 6/6 |  | Fine and medium sands (subangular quartz, subrounded/rounded micritic and bioclastic limestone and feldspar; zircon and tourmaline inclusions); some mineral grains present ferruginous coatings (inherited feature);  Massive microstructure; c/f related distribution chitonic-enaulic; crystallistic b-fabric;  OM: absent / residual;  Pedofeatures: calcitic coatings, nodules and concretions; Mn-Fe orthic nodules; |
| **SU 625** | | |
| UNIT-IV  AR-625  Dry: 7.5.YR 6/4; Wet: 7.5.YR 5/4 | Conical structure is located in the north-eastern corner of SU 604 (0.32m long x 0.18m wide x 0.25m deep); 0.40 m long limestone block, thrust in at a 45º angle; | Fine and medium sands (subangular quartz, subrounded/rounded micritic and bioclastic limestone and feldspar; zircon and tourmaline inclusions); some mineral grains present ferruginous coatings (inherited feature);  Massive microstructure; c/f related distribution chitonic-enaulic; crystallistic b-fabric;  OM: scarce reddish patches (mineralized) and charcoal (sand-silt sized);  Pedofeatures: calcitic coatings, nodules and concretions; Mn-Fe orthic nodules; |
| **SU-613** | | |
| UNIT III / IV  AR-610-2-top (next to the hearth-pit)  AR-610-2-bottom (next to the hearth-pit) | Irregular morphology (1.17 m long X 1.08m wide X 0.17 deep); rock fragments in the cobble size range are randomly distributed, although some superpositions were observed in the field; | Fine and medium sands (subangular quartz) and feldspar; zircon and tourmaline inclusions); random calcitic sands; some mineral grains present ferruginous coatings (inherited feature);  Massive microstructure; combination of c/f related distribution coarse monic – chitonic; speckle b-fabric and c/f related distribution chitonic-enaulic and crystallistic b-fabric;  Lack of calcitic pedofeatures, mineralized OM, plant tissue and biogenic activity traits;  OM: random sand-size charcoal grains; residual silt-size charcoal;  Pedofeatures: random calcitic coatings and concretions; Mn-Fe orthic nodules; |
| UNIT IV  AR-613-1 top (south area)  Dry: 7.5.YR 5/2; Wet: 7.5.YR 4/4  AR-613-1-bottom (south area)  Dry: 7.5.YR 5/4; Wet: 7.5.YR 4/4  AR-613-2 -top (central area)  Dry: 7.5.YR 6/2; Wet: 7.5.YR 5/2-5/4  AR-613-2-bottom (central area)  Dry: 7.5.YR 6/2; Wet: 7.5.YR 5/2-5/4  AR-613-2-base (central area)  Dry: 7.5.YR 6/2; Wet: 7.5.YR 5/2-5/4  AR-613-0 (grey) (central area)  Dry: 7.5.YR 6/4; Wet: 7.5.YR 5/4  AR-613-1 (grey) (central area)  Dry: 7.5.YR 6/4; Wet: 7.5.YR 5/4  AR-613-2 (central area)  Dry: 7.5.YR 6/4; Wet: 7.5.YR 5/4 |  | AR-613-3 (central area)  Dry: 7.5.YR 5/2-4/2; Wet: 7.5.YR 4/2-4/4  AR-613-4 (central area)  Dry: 7.5.YR 5/2-4/2; Wet: 7.5.YR 4/2-4/4  AR-613-5 (central area)  Dry: 7.5.YR 5/2-4/2; Wet: 7.5.YR 4/2-4/4  AR-13-6 (central area)  Dry: 7.5.YR 5/2-4/2; Wet: 7.5.YR 4/2-4/4  AR-613-7 (central area)  Dry: 7.5.YR 5/2-4/2; Wet: 7.5.YR 4/2-4/4  AR-613-8 (central area)  Dry: 7.5.YR 5/2-4/2; Wet: 7.5.YR 4/2-4/4  AR-610-1-top (north area)  Dry: 7.5.YR 5/4; Wet: 7.5.YR 4/4  AR-610-1-bottom (north area)  Dry: 7.5.YR 5/4; Wet: 7.5.YR 4/4 |
| **SU-615** | | |
| UNIT IV  AR-615  Dry: 7.5.YR 5/4  Wet: 7.5.YR 4/4 | Subcircular (1.16m long X 0.92m wide X 0.25m deep); random carbonate rock fragments; | Fine and medium sands (subangular quartz, subrounded/rounded micritic and bioclastic limestone and feldspar; ash pseudomorphs, zircon and tourmaline inclusions); some mineral grains present ferruginous coatings (inherited feature);  Massive microstructure; c/f related distribution chitonic-enaulic locally vughy, with channels and planes; crystallistic b-fabric;  OM: reddish patches (mineralized); charcoal (sand-silt sized); plant tissue and root sections;  Pedofeatures: calcitic coatings, nodules and concretions; acicular calcite; enchytraeid excrements; Mn-Fe orthic nodules; |
