# Supplementary material for: Mesolithic hearth-pits and formation processes: a geoarchaeological investigation of sediments from El Arenal de la Virgen site (SE Iberia)
Source: Archaeol Anthropol Sci. 2023 Jun 22;15(7):104. doi: 10.1007/s12520-023-01794-5 (PMC10287818; doi:10.1007/s12520-023-01794-5)
Supplement: Supplementary file 2 — Supplementary file2 (DOC 95 KB) [file 12520_2023_1794_MOESM2_ESM.doc]

| **SU 604** | | | | | | | | | | | | | | |
| --- | --- | --- | --- | --- | --- | --- | --- | --- | --- | --- | --- | --- | --- | --- |
| UNIT | SAMPLE ID | pH | OM  % | CaCO3  % | CLAY  % | SILT-F  % | SILT-C  % | SAND –VF  % | SAND -VF  % | SAND-F  % | SAND-M  % | SAND-C  % | SAND-VC % | TEXTURE |
| III /IV | B-604-1 | 8,76 | 0,96 | 5 | 6,8 | 2,3 | 1,0 | 0,6 | 3,0 | 56,3 | 28,5 | 1,4 | 0,1 | Sandy |
| IV | B-604-2 | 8,72 | 1,08 | 8 | 6,3 | 0,3 | 0,3 | 0,9 | 2,7 | 50,7 | 37,2 | 1,5 | 0,1 | Sandy |
| IV | B-604-3 | 8,73 | 1,10 | 10 | 6,9 | 2,6 | 0,5 | 0,7 | 3,4 | 62,3 | 22,2 | 1,3 | 0,1 | Sandy Loam |
| IV | B-604-4 | 8,74 | 0,98 | 9 | 6,8 | 1,8 | 0,5 | 0,5 | 2,4 | 54,5 | 31,7 | 1,6 | 0,2 | Sandy Loam |
| IV | B-604-5 | 8,78 | 0,93 | 10 | 2,2 | 2,5 | 1,7 | 0,9 | 3,7 | 65,4 | 22,3 | 1,2 | 0,1 | Sandy |
| IV | B-604-6 | 8,77 | 0,94 | 10 | 5,9 | 1,5 | 1,3 | 0,8 | 2,9 | 65,6 | 20,7 | 1,2 | 0,1 | Sandy |
| V | B-604-7 | 8,76 | 0,88 | 10 | 6,2 | 0,8 | 1,0 | 0,8 | 4,3 | 64,0 | 21,5 | 1,2 | 0,2 | Sandy |
| V | B-604-8 | 8,74 | 0,86 | 10 | 5,1 | 0,8 | 0,8 | 0,7 | 4,6 | 67,3 | 19,5 | 1,1 | 0,1 | Sandy |
| V | B-604-9 | 8,79 | <0,50 | 10 | 5,4 | 0,5 | 0,8 | 0,7 | 3,8 | 61,6 | 25,9 | 1,1 | 0,2 | Sandy |
| V | B-604-10 | 8,79 | <0,50 | 10 | 4,9 | 0,5 | 1,1 | 0,9 | 2,3 | 61,9 | 26,6 | 1,6 | 0,2 | Sandy |
|  |  |  |  |  |  |  |  |  |  |  |  |  |  |  |
| **SU 625** | | | | | | | | | | | | | | |
| IV | B-625-1-top | 8,69 | 0,53 | 9 | 6,7 | 1,9 | 3,2 | 0,8 | 0,8 | 53,8 | 28,6 | 1,5 | 0,2 | Loamy Sand |
| IV /V | B-625-1-bott | 8,72 | <0,50 | 8 | 5,9 | 1,8 | 1,3 | 0,7 | 3,4 | 63,1 | 22,0 | 1,6 | 0,2 | Sandy |
|  |  |  |  |  |  |  |  |  |  |  |  |  |  |  |
| **SU 613** | | | | | | | | | | | | | | |
| IV | B-613-1 top | 8,72 | 0,82 | 11 | 7,9 | 2,5 | 1,9 | 1,2 | 2,4 | 50,4 | 30,4 | 2,7 | 0,6 | Loamy Sand |
| IV | B-613-1-bott | 8,71 | 0,74 | 13 | 8,1 | 2,3 | 2,9 | 0,9 | 3,0 | 45,7 | 34,5 | 2,2 | 0,4 | Loamy Sand |
| IV | B-613-2-top | 8,65 | 0,81 | 15 | 7,0 | 2,3 | 4,7 | 1,4 | 3,8 | 48,9 | 29,8 | 1,7 | 0,4 | Loamy Sand |
| IV | 613-2-bott | 8,70 | 0,75 | 15 | 4,4 | 2,1 | 6,4 | 1,6 | 4,0 | 58,7 | 20,4 | 2,1 | 0,3 | Sandy |
| IV | B-613-2-base | 8,62 | 0,65 | 13 | 5,3 | 2,3 | 4,0 | 1,3 | 3,5 | 61,0 | 20,9 | 1,5 | 0,2 | Sandy |
| IV/III | B-613-0 | 8,73 | <0,50 | 3 | 3,9 | 1,1 | 1,1 | 0,8 | 3,0 | 54,1 | 34,5 | 1,1 | 0,4 | Sandy |
| IV | B-613-1 (GR) | 8,75 | 0,53 | 6 | 5,4 | 1,3 | 1,9 | 0,9 | 4,8 | 72,4 | 11,8 | 1,4 | 0,1 | Sandy |
| IV | B-613-2 | 8,68 | 0,64 | 9 | 6,0 | 2,1 | 2,3 | 0,8 | 2,4 | 65,4 | 19,3 | 1,5 | 0,2 | Sandy |
| IV | B-613-3 | 8,70 | 0,64 | 10 | 6,8 | 2,3 | 3,4 | 1,0 | 3,8 | 59,1 | 22,0 | 1,4 | 0,2 | Sandy |
| IV | B-613-4 | 8,69 | 0,69 | 11 | 5,8 | 2,5 | 4,0 | 1,1 | 3,1 | 61,6 | 19,9 | 1,9 | 0,1 | Sandy |
| IV | B-613-5 | 8,66 | 0,80 | 13 | 6,4 | 7,2 | 0,5 | 1,1 | 2,5 | 55,0 | 24,8 | 2,2 | 0,3 | Loamy Sand |
| IV | B-613-6 | 8,67 | 0,81 | 14 | 5,5 | 1,8 | 5,0 | 0,7 | 2,3 | 50,2 | 32,0 | 2,2 | 0,3 | Sandy |
| IV | 613-7 | 8,68 | 0,75 | 15 | 5,2 | 2,8 | 3,9 | 1,2 | 3,4 | 58,7 | 22,7 | 1,9 | 0,2 | Sandy |
| IV | B-613-8 | 8,68 | 0,68 | 12 | 7,5 | 2,7 | 3,2 | 1,5 | 2,5 | 39,9 | 39,4 | 2,7 | 0,6 | Loamy Sand |
| IV | B-610-1-top | 8,65 | 0,64 | 10 | 6,6 | 0,3 | 3,8 | 0,6 | 2,9 | 60,0 | 24,2 | 1,4 | 0,2 | Sandy |
| IV | B-610-1-bott | 8,67 | 0,62 | 11 | 4,0 | 1,1 | 1,6 | 0,9 | 3,3 | 66,0 | 21,2 | 1,8 | 0,1 | Sandy |
| III  (next to hearth-pit) | B-610-2-top | 8,70 | 0,57 | 5 | 5,6 | 1,5 | 1,3 | 0,5 | 2,7 | 66,1 | 20,9 | 1,3 | 0,1 | Sandy |
| III/IV  (next to hearth-pit) | B-610-2-bott | 8,66 | 0,63 | 9 | 7,8 | 1,1 | 0,3 | 0,6 | 3,0 | 56,3 | 29,2 | 1,5 | 0,2 | Sandy |
