# Supplementary material for: Mesolithic hearth-pits and formation processes: a geoarchaeological investigation of sediments from El Arenal de la Virgen site (SE Iberia)
Source: Archaeol Anthropol Sci. 2023 Jun 22;15(7):104. doi: 10.1007/s12520-023-01794-5 (PMC10287818; doi:10.1007/s12520-023-01794-5)
Supplement: Supplementary file 3 — Supplementary file3 (DOC 69 KB) [file 12520_2023_1794_MOESM3_ESM.doc]

|  | | |
| --- | --- | --- |
|  | **MACROSCOPIC** **FEATURES** | **MICROSCOPIC** **FEATURES** |
| **CARBONATE ROCKS - EL ARENAL-HEARTH-PITS** |  | |
| PHASE 1  SU 608; SU 611  (Field data + micromorphological thin sections + petrographic thin section SU 608 + petrographic thin section SU 611) | Subangular and subrounded morphology; rounded edges; *in situ* deep fissures and surface cracks in combination with light-weight and friable inner matrix in some specimens; grey-dark grey colour with occasional black and red patches; calcitic concretions on surface; high porosity associated to dark areas and brittle cortex in some specimens;  SU 608: accumulation of burnt carbonate rocks; layered pattern; relatively homogeneous cobble size at the base of the structure; SU 611: lack of rock fragments inside the dark sediment of the structure but concentrated on the outer side of the hearth-pit; | SU 608; light brown grey porous cortex with small oxide inclusions; micritic-sparitic matrix light grey in PPL and XPL; matrix fossils showed reddish brown shade and significant darker grey colour; light yellow-grey clayey coating around the cortex with silt-size charcoal inclusions and isotropic in XPL;  SU 611: bioclastic porous matrix; darkening of matrix fossils; slight brown-reddening of the matrix; slight oxide concentration; calcitic recrystallization of matrix pores. |
| PHASE 2  SU 604; SU 625; SU 613  (Field data + micromorphological thin sections + petrographic thin section SU 604 + petrographic thin section SU 613)) | Subangular and subrounded morphology; rounded edges; *in situ* deep fissures and surface cracks in combination with light-weight and friable inner matrix in some specimens; grey-dark grey colour with occasional black and red patches; calcitic concretions on surface; high porosity associated to dark areas and brittle cortex in some specimens;  SU 604: carbonate rock assemblage randomly distributed; relatively high size variability (i.e., pebble-boulder size); SU 625: a 0.40 m long limestone block, thrust in at a 45º angle; SU 613: carbonate rock fragments in the small cobble size range; randomly distributed; some superpositions observed;  SU 615: random carbonate rock fragments; cobble size range; | SU 604: bioclastic, micritic and sparitic porous matrix; grey-brown porous cortex; clayey yellow-grey isotropic coatings with silt-size microcharcoal embedded; darkening of calcitic fossils and pore walls to a brown-grey colour; dark grey colouring of fungus hyphae; fissures showing dark grey-black coatings and dark grey sediment inclusions; calcitic recrystallizations inside matrix pores.  SU 613: bioclastic, micritic and sparitic porous matrix; compacted clayey yellow-grey ashy coating; greyish/reddish brown fungus hyphae; slight grey-brown shade on the matrix; grey-brown coatings around pores and fissures; calcitic recrystallizations around pores; |
| **REFERENCE CARBONATE ROCKS–EL CASTELLAR OUTCROP (FRESH / UNBURNED)** |  | |
| GROUP 1  (N=11; field data + 6 petrographic thin sections) | Limestone; subangular, rounded edges; ≤10 cm max. side; beige colour with dark grey areas; parcial or total porous calcitic cortex (≤ 1 cm thick) from physico-chemical weathering;  Sample ID: Ref. 2, Ref. 3, Ref. 4, Ref. 5, Ref. 7, Ref. 8, Ref. 11, Ref. 17, Ref. 19, Ref. 20, Ref. 21; | Sample ID: Ref. 2, Ref. 5, Ref. 7, Ref. 8,  Ref. 13, Ref. 19;  Cortex: light grey and highly birefringent sparitic domains; grey brown low birefringent micritic domains; porous; fissures with greyish brown coatings; fissures with calcitic recrystallizations; micritic layering;  Matrix: darker grey in colour when compared to the cortex; porous; fossils embedded in micritic-sparitic matrix; |
| GROUP 2  (N=5; field data) | Limestone; subangular; rounded edges; ≤10 cm max. side; beige with dark grey areas; porous calcitic cortex + depressions (≤ 1 cm wide), in some cases interconnected, from physico-chemical water-related weathering;  Sample ID: Ref. 10, Ref. 13, Ref. 15, Ref. 24, Ref. 25; | XXX |
| GROUP 3  (N=7; field data + 3 petrographic thin sections) | Calcitic crust; subangular; rounded edges; ≤10 cm max. side; light beige with dark grey areas; rounded millimetric pores covering the specimens on both sides;  Sample ID: Ref. 1, Ref. 6, Ref. 9, Ref. 12, Ref. 16, Ref. 22, Ref. 23; | Sample ID: Ref. 1, Ref. 6, Ref. 9;  Laminar microstructure alternating layers of highly birefringent light grey sparite and low birefringent greyish brown micrite; high porosity; |
| GROUP 4  (N=2; field data + 2 petrographic thin sections) | Mudstone, subangular-subrounded, rounded, smooth edges; grey-blackish colour; porous; milimetric rounded depressions from physico-chemical erosion.  Sample ID: Ref. 14, Ref. 18 | Sample ID: Ref. 14; Ref. 18;    Dark grey colour; highly porous; mainly micritic; greyish/reddish brown fungus; |
| **EXPERIMENTAL BURNING CARBONATE ROCKS** |  | |
| GROUP 1  Ref. 8 (sectioned specimen)  (N=4; 4 petrographic thin sections) | . | |
| PRE-COMBUSITON 0ºC | Light beige on both sides; fissures on both sides; white-orange weathering cortex 1 cm thick approx.; grey crystalline vein with mineral luster on one of the sides of the sample; | Cortex: micritic and sparitic light grey alternating grey brown areas; porous; fissures with greyish brown coatings; fissures with calcitic recrystallization;  Matrix: darker grey in colour when compared to the cortex; porous; fossils embedded in micritic-sparitic matrix; |
| 300-350ºC | Beige-grey on both sides; the light beige colour of the cortex turns into light grey, while the orange turns into very dark grey-black; no changes observed on the fissural pattern;  Weight loss: 0.2 g (2%) | Cortex: local darkening and intensification of browning; calcite crystals show similar size, shape and crystallinity than those observed on the unburned sample;  Matrix: fossils show reddish brown shade and significant darker grey colour; random oxide concentrations; |
| 500-550ºC | Darker grey colour covering the sample completely; dark orange areas locally; intensified grey colour on the cortex ; the crystalline vein becomes whitish and dull; intensification of preexisting fissures resulting in fragmentation of the outer portion of the sample following a preexisting fissure; Weight loss: 0.5 g (1%). | Cortex: light brown-grey colour shade, which is observed on most calcite crystals in contrast to 300-350ºC sample; micritic and sparitic calcite still clearly observed; random blackish mineral patches dull in PPL and isotropic in XPL increased porosity;  Matrix: light reddish-brown calcite cement; dark grey bioclasts; frequent oxide features (e.g., nodules, coatings and impregnations);  Intensification of fissures; |
| 700-750ºC | Homogeneous white colour all over the sample; general light orange shade with intensification of orange colour on depressions and cracks; the cortex shows intensification of the whitish and orange-reddish colours; intensification of preexisting fissures; the sample however, holds together; Weight loss: 1.6 g (5%) | Cortex: dark grey-blackish in PPL; substantial loss of matter;  Matrix: calcite cement very dark grey, isotropic and dull; only random fossils discernible; increased porosity following a vesicular pattern; intensification and deepening of fissures; oxides less visible; |
| GROUP 4  Ref. 14 (sectioned specimen)  (N=4; 4 petrographic thin sections) |  |  |
| PRE-COMBUSTION 0ºC | Grey colour of the sample with a darker areas on the outer rim; millimetric pores all over the sample and on both sides of the specimen; small fissures; | Dark grey colour; highly porous; mainly micritic; greyish/reddish brown fungus hyphae especially concentrated in the outer rim; |
| 300-350ºC | Colour homogenization of the sample towards light grey; no visible alteration of the fissural pattern;  Weight loss: 0.1 g (2%) | Grey colour; calcite cement appear unchanged; greyish/reddish brown fungus hyphae; |
| 500-550ºC | Light grey colour with an orange shade uniformly covering all the sample; no visible alteration of the fissural pattern;  Weight loss: 0.2 g (4%). | Calcite cement does not show evident changes; fungus hyphae turn more grey and dull; development of oxide features significantly lower compared to bioclastic limestone; calcitic recrystallizations in pores; |
| 700-750ºC | Homogeneous white colour thorogouh the sample; small orange-reddish concentrations on the pores and depressions; blueish-grey area on the cortex; no visible alteration of the fissural pattern; chalky texture (white dust on the fingers after holding the sample);  Weight loss: 1.1 g (12%) | Calcite cement does not show substantial changes; similar porosity pattern compared to lower combustion temperatures; dull grey fungus hyphae; fine fissures; |
| GROUP 1  Ref. 4 (whole specimen)  N=1 |  | XXX |
| PRE-COMBUSTION 0ºC | Dark grey cortex with light brown – orange ridges; dark beige with mineral luster on the side lacking cortex; |  |
| 300-350ºC | Orange-brown colour on both sides; the side lacking cortex looks duller; no visible alteration of the fissural pattern;  Weight loss: 0.7 g (0.5%) |  |
| 500-550ºC | The cortex turns into intense orange colour and the ridges become grey; the rest of the sample turns into grey with dark orange patches; development of fissures on both sides from thermal impact;  Weight loss: 0.6 g (0.3%) |  |
| 700-750ºC | Right after furnace removal: lighter orange colour of the cortex and whitish ridges with smother edges; brittle texture; the side lacking cortex turns into whitish with brighter orange patches; increased and deepened fissural pattern from thermal impact;  After 3 days of exposure to laboratory conditions (23ºC- 70% RH approx.): cracked and flaky cortex; mostly reddish in colour from oxide concentration; white inner matrix, chalky, deeply fissured, very fragile;  Weight loss: 8 g (0.6%) |  |
|  |  |  |
